# Roles of Traditional and Next-Generation Probiotics on Non-Alcoholic Fatty Liver Disease (NAFLD) and Non-Alcoholic Steatohepatitis (NASH): A Systematic Review and Network Meta-Analysis

**DOI:** 10.3390/antiox13030329

**Published:** 2024-03-07

**Authors:** Yuezhi Zhu, Jen Kit Tan, Jia Liu, Jo Aan Goon

**Affiliations:** 1 Department of Biochemistry, Faculty of Medicine, Universiti Kebangsaan Malaysia, Kuala Lumpur 56000, Malaysia; 2 Department of Nursing, Faculty of Medicine, Universiti Kebangsaan Malaysia, Kuala Lumpur 56000, Malaysia

**Keywords:** NAFLD/NASH, traditional probiotics, next-generation probiotics (NGPs), short-chain fatty acids (SCFAs), antioxidant

## Abstract

Non-alcoholic fatty liver disease (NAFLD) and its progressive stage, non-alcoholic steatohepatitis (NASH), are becoming one of the most common chronic liver diseases globally. Lifestyle interventions such as weight reduction, increased physical activity, and maintaining healthy diets play a pivotal role in managing NAFLD/NASH. Recent studies suggest that the gut microbiome is associated with the pathogenesis of NAFLD/NASH, prompting microbiome-targeted therapy to emerge as a new therapeutic option for NAFLD/NASH. We conducted a systematic review based on the PRISMA statement and employed network meta-analysis to investigate the effects of traditional probiotics and next-generation probiotics (NGPs) on NAFLD/NASH. Comparative analysis reveals that traditional probiotics primarily reduce liver fat deposition and inflammation by improving gut microbiota composition, enhancing intestinal barrier function, and modulating immune responses. In contrast, NGPs demonstrate a more significant therapeutic potential, attributed to their direct effects on inhibiting oxidative stress and their ability to enhance the production of short-chain fatty acids (SCFAs), NGPs appear as a new potential strategy for the management of NAFLD/NASH through their dual action of directly inhibiting oxidative stress and enhancing SCFA production, highlighting the importance of understanding and utilizing the direct and indirect regulatory mechanisms of oxidative stress in the management of NAFLD/NASH.

## 1. Introduction

Non-alcoholic fatty liver disease (NAFLD) is a prevalent chronic liver condition, with 10–20% of patients progressing to non-alcoholic steatohepatitis (NASH) [1]. NAFLD/NASH often coexists with metabolic disorders such as obesity, diabetes, and dyslipidemia, mutually influencing each other [2]. While lifestyle changes are the primary approach for managing NAFLD/NASH, there are practical challenges that hinder their implementation. These challenges include the difficulty of maintaining a healthy lifestyle, slow manifestation of effects, and the inability of some patients to achieve ideal treatment outcomes through lifestyle modifications. These factors can potentially limit the long-term sustainability and effectiveness of lifestyle changes as the sole treatment approach [3,4]. Recognizing these limitations, it is particularly important to actively explore and develop alternative and supplementary treatment strategies. By targeting the specific pathophysiological mechanisms involved in NAFLD/NASH, such as lipid accumulation, oxidative stress, inflammatory responses, and fibrosis, integrating pharmacological interventions and adjunctive therapies can lead to more targeted and expedited treatment outcomes [3,4]. It is important to emphasize that these therapeutic approaches should not be considered substitutes for lifestyle modifications but rather complementary strategies that should be implemented concurrently [3,4]. Treatment for NAFLD/NASH should adopt a comprehensive management plan, encompassing a holistic and integrated approach, to effectively control and address the condition, ultimately improving patient outcome [3,4]. Recently, gut microbiota imbalance has gained attention in association with NAFLD/NASH [5]. An imbalanced gut microbiome may lead to impaired gut barrier function and increased intestinal permeability, causing liver inflammation and damage [5,6]. Therefore, regulating the imbalanced gut microbiota using various types of probiotics has become an emerging therapeutic strategy aimed at restoring balance and improving histological conditions [6,7].

Probiotics are beneficial microorganisms for the human body, possessing a diverse range of physiological functions, including the inhibition of pathogenic bacteria and cholesterol degradation [8]. Well-established probiotics such as lactic acid bacteria and bifidobacteria primarily regulate gut flora balance and enhance intestinal immunity through oral administration, thereby effectively controlling disease progression [8,9]. Complementing these are prebiotics, non-digestible food components that serve as nourishment for probiotics, promoting their growth and activity [7]. Synbiotics combine both probiotics and prebiotics to synergistically maintain gut microbiota balance more efficiently [7,10]. Together, these constituents constitute the traditional probiotic system, which ensures gut health and equilibrium through intricate interactions. Next-generation probiotics (NGPs) are typically identified through advanced methods such as high-throughput sequencing and bioinformatics analysis. This approach enables the discovery of previously unrecognized probiotic strains that possess unique health-promoting characteristics and mechanisms of action [11,12]. Unlike traditional probiotics, NGPs are characterized by their specific and diverse mechanisms, tailored to address various disease conditions. These mechanisms include but are not limited to the modulation of the gut-brain axis, gut-liver axis, enhancement of the immune system, alteration of metabolic pathways, and direct antagonistic effects against pathogens [11,13]. With advancements in technology and the emergence of microbiomics research, NGPs have gained significance. The mechanisms underlying NGP actions are multifaceted, encompassing interactions with host cells, modulation of immune responses, and production of bioactive substances [11,13]. Moreover, NGPs place greater emphasis on personalized treatment by selecting suitable probiotics based on individualized characteristics of each patient’s gut microbiome composition [14]. This paradigm shift signifies a transition in probiotic research from conventional nutritional supplementation towards a more precise medical approach.

In recent years, NGPs have demonstrated significant therapeutic potential in the management of NAFLD/NASH [15]. The mechanisms underlying the efficacy of NGPs extend beyond gut microbiota modulation and encompass direct modulation of hepatic oxidative stress, lipid metabolism, and inflammatory responses [16]. By generating bioactive compounds such as short-chain fatty acids (SCFAs), NGPs can indirectly ameliorate hepatic metabolic dysregulation, mitigate oxidative stress in hepatocytes, and alleviate chronic inflammation resulting from gut microbiota imbalances [17]. Moreover, NGPs can indirectly modulate signaling pathways associated with oxidative stress in hepatocytes through alterations in bile acid composition [18]. NGPs can directly or indirectly participate in antioxidant mechanisms and affect liver metabolic pathways, thereby contributing to improved liver inflammation and lipid metabolism.

Oxidative stress plays a pivotal role in the pathogenesis and progression of NAFLD/NASH [19]. The accumulation of excessive fatty acids in hepatocytes triggers oxidative stress, which is further exacerbated by insulin resistance, obesity, and metabolic syndrome [20]. An imbalance between the generation of reactive oxygen species (ROS) and the liver’s capacity to detoxify these reactive intermediates or repair resultant damage activates a cascade of signaling pathways [20,21]. The NF-κB and MAPK pathways amplify the inflammatory response, while the crosstalk between PI3K/AKT and NF-κB governs cell survival and inflammation [22]. The TGF-β/Smad pathway centrally contributes to fibrosis and is influenced by PI3K/AKT signaling, impacting hepatic stellate cell activation and collagen production [22]. Additionally, the JAK/STAT pathway is an indispensable component of both inflammatory and fibrotic responses, interacting with NF-κB and MAPK pathways [22,23]. These interconnected signaling cascades underscore the intricate pathophysiology of NAFLD/NASH under conditions of oxidative stress. Given that oxidative stress serves as a key driving force in NAFLD/NASH progression, mitigating its effects can not only reduce liver inflammation but also diminish the risk of hepatocellular damage and fibrosis.

The number of studies exploring the therapeutic potential of traditional probiotics and NGPs for NAFLD/NASH is increasing rapidly, necessitating a more accurate assessment of their relative effectiveness and tolerance. Traditional meta-analyses often employ estimation methods when comparing therapeutic effects, making it challenging to determine the precise efficacy of these interventions. By adopting a paired problem approach, we can better evaluate the effects of these interventions. Therefore, we present a systematic review and network meta-analysis to collect and summarize research findings on treatments based on traditional probiotics and NGPs in clinical and preclinical studies of NAFLD/NASH with the aim of evaluating their overall therapeutic efficacy. This systematic approach allows for a comprehensive comparative assessment of the efficacy between traditional probiotics and NGPs for managing NAFLD/NASH.

The findings of this review offer insights to healthcare professionals regarding the potential role of probiotics, encompassing both traditional ones and NGPs, as adjunctive therapies for managing NAFLD/NASH. Understanding the efficacy and safety profiles of various probiotic strains can help clinicians make informed treatment decisions for their patients. Future studies could further investigate how probiotics work in NAFLD/NASH, as well as optimal dosing and long-term effects. Comparative analyses among different probiotic strains and formulations could refine treatment protocols and identify the most effective interventions for NAFLD/NASH patients. Ultimately, ongoing research in this field holds promise for enhancing patient outcomes and developing more personalized approaches to managing NAFLD/NASH.

## 2. Materials and Methods

To investigate the therapeutic effects of traditional probiotics and NGPs on NAFLD/NASH, our study adheres to the guidelines and principles outlined in the Preferred Reporting Items for Systematic Reviews and Meta-Analyses (PRISMA) statement. Furthermore, our predefined review protocol was prospectively registered in the International Prospective Register of Systematic Reviews (PROSPERO) with registration number CRD42023445898.

### 2.1. Search Strategy

The literature search was restricted to English articles retrieved from the databases PubMed, Embase, Web of Science, Cochrane, and Medline. The search strategy employed Medical Subject Headings (MeSH) and keywords such as “probiotic”, “prebiotic”, “synbiotic”, “next-generation probiotics (NGPs)”, “NAFLD”, “NASH”, “non-alcoholic fatty liver disease”, “non-alcoholic steatohepatitis”, “steatosis”, and combinations of these MeSH terms. Furthermore, a manual search was conducted on the references of all original articles, liver conference reports, abstracts, and posters to gather relevant research findings available until 20 November 2023.

### 2.2. Inclusion and Exclusion Criteria

The inclusion criteria for this study encompassed randomized controlled trials (RCTs) in clinical settings as well as animal models of NAFLD/NASH induced by a high-fat diet (HFD) or other standardized methods. Additionally, clinical studies had to incorporate measurements of alanine aminotransferase (ALT), aspartate aminotransferase (AST), triglycerides (TGs), total cholesterol (TC), fasting blood glucose (FBG), or results from imaging studies. Furthermore, only English-written articles are included in this review.

Excluded from this study were cases of liver steatosis induced by other causes such as alcoholic hepatitis, viral hepatitis, genetic disorders, etc. Moreover, studies with unavailability of outcome measures for meta-analysis were also excluded. Publications that fall under categories like reviews, case reports, and conference abstracts were not included.

### 2.3. Data Extraction

Two investigators (YZ.Z. and J.L.) independently screened the titles and abstracts of each study. In cases of disagreement regarding the selection of studies, consultations were arranged, and potential discrepancies were resolved through open discussions with other investigators (J.A.G and J.K.T.). The variables considered for preclinical studies included the following: (1) Study Characteristics: Title, authors, publication year, study location, and sample size. (2) Experimental Model Characteristics: Lineage, number of animals per group, gender, age, and the NAFLD/NASH induction protocol. (3) Intervention Scheme: Types of probiotics/NGPs used, the dosage administered, and the timing of administration. (4) Post-intervention outcomes assessed. (5) Study objective determined. For clinical studies, the variables considered for intervention outcomes were as follows: (1) Study Characteristics: Title; authors’ names listed in full with initials only if applicable; publication year; study location; sample size specified clearly. (2) Population Characteristics: Gender distribution is provided along with the age range mentioned; the total number of participants is indicated precisely. (3) Experimental Design Details: Randomization process described explicitly; presence or absence of a placebo-controlled group stated clearly; double-blind methodology employed when applicable mentioned explicitly as well. (4) Intervention Scheme: Types of probiotics/NGPs, dosage, timing of administration, frequency of administration. (5) Post-intervention outcomes. (6) Study objective.

### 2.4. Quality Assessment

To ensure the reliability of the included studies, we assessed the risk of bias in the RCTs using the Cochrane-recommended ROB 2 tool. The evaluation of these studies was based on their utilization of Intention-to-Treat (ITT) or Per-Protocol (PP) analysis methods. For studies employing ITT analysis, we evaluated how they addressed non-compliance and missing data, as well as their potential impact on study outcomes. In the case of PP analysis, our focus was on assessing the strictness of exclusion criteria and their potential influence on selection bias. Studies exhibiting a high risk of bias in one domain or some concerns in three domains during the ROB 2 assessment were classified as having a high risk of bias. Regarding preclinical study reports, SYRCLE’s RoB tool was employed for assessing bias risk; if a study demonstrated a high risk of bias in one domain during ROB assessment, it was considered to have a high risk of bias.

### 2.5. Outcome Measurement

The main outcome of our study was the change in serum ALT concentration, and the secondary outcomes were the mean change in serum AST, serum TGs, serum TC, serum glutamyltransferase (GGT), FBG, Fibroscan Controlled Attenuation Parameter Score (Fibroscan CAP score), and body mass index (BMI).

### 2.6. Statistic Analysis

A standard meta-analysis was conducted using R-Studio software (version 4.2.2). The mean difference (MD) and 95% confidence interval (CI) were calculated for each parameter. Heterogeneity among studies was assessed using the Q test and I^2^ statistic. If the Q test yielded a *p*-value greater than 0.05 and I^2^ value less than or equal to 50%, a fixed-effect model was employed for the analysis; otherwise, a random-effects model was utilized. Baujat Plot and Influence Characteristics methods were applied to identify and exclude outliers. In cases of heterogeneity, subgroup analysis and meta-regression were performed to explore the sources of heterogeneity among different studies. Subgroup analysis variables included differences in country and region based on the characteristics of the study population. Meta-regression identified potential sources of heterogeneity, considering factors such as colony-forming units (CFU) in treatment, baseline ALT and AST levels, and patient age. To avoid missing significant factors, the significance level α was relaxed to 0.1.

For network meta-analysis using R-Studio software, a network plot illustrating relationships between different interventions was generated by depicting circles whose sizes represented sample sizes of interventions while line thickness indicated a number of comparative studies between two interventions. The netmeta package in R-Studio software facilitated conducting network meta-analysis with subsequent generation of forest plots. Effect size for binary data is expressed as odds ratio (OR) along with its corresponding 95% CI, whereas for continuous data, it is expressed as MD accompanied by its respective 95% CI. Probability ranking based on *p*-values determined the best outcomes. Funnel plots, along with Egger’s linear regression, were employed to assess publication bias.

## 3. Results

### 3.1. Search Result

**Figure 1:**
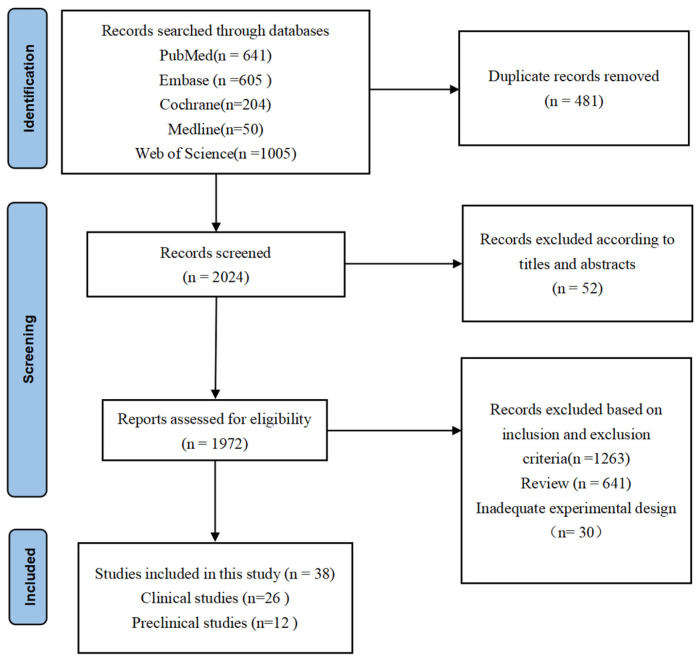
PRISMA flowchart. The final 38 research studies that met the criteria were included in the qualitative synthesis based on the number of titles, abstracts, and whole texts screened.

A total of 2505 papers were identified through comprehensive literature searches in various databases. Among these publications, 533 papers were excluded after careful evaluation of titles and abstracts due to duplicate records and non-compliance with the predefined inclusion criteria. The remaining 1972 articles underwent a thorough full-text review process. Subsequently, a total of 1934 articles were excluded for diverse reasons: 1263 were omitted based on the specified inclusion and exclusion criteria, 641 were categorized as reviews, and 30 exhibited inadequate experimental design. Ultimately, only 38 [24,25,26,27,28,29,30,31,32,33,34,35,36,37,38,39,40,41,42,43,44,45,46,47,48,49,50,51,52,53,54,55,56,57,58,59,60,61] articles fulfilled all eligibility criteria and thus were included in this systematic review. The detailed search strategy and article selection process are concisely summarized in Figure 1.

### 3.2. Characteristics of Included Study

In a total of 26 [24,25,26,27,28,29,30,31,32,33,34,35,36,37,38,39,40,41,42,43,44,45,46,47,48,49] clinical studies involving 1625 participants, probiotic treatment (*Bifidobacterium*, *Lactobacillus*, *Streptococcus*, *Clostridium butyricum*) was analyzed. Among these studies, 25 focused on traditional probiotics (*Bifidobacterium*, *Lactobacillus*, *Streptococcus*). Of the included studies, 16 were conducted in Asian countries, while the remaining 10 originated from non-Asian countries (7 from Europe and 3 from the Americas). The age range of participants was broad (18–75 years), encompassing both genders. Most of these studies followed a double-blind, randomized, placebo-controlled crossover design, with some adopting a parallel design. The dosage of probiotics and synbiotics varied significantly across studies, ranging from several tens of 10^7^ to 10^9^ of CFU per day. Intervention periods were also variable, with some lasting for 4 weeks while others extended to durations such as 12 weeks or even longer. The primary objectives addressed in these studies were evaluating the impact of probiotics and synbiotics on liver function, blood lipid levels regulation, inflammatory markers modulation, gut microbiota composition alteration, insulin resistance mitigation, and hepatic fat content. Additionally, some studies also indicate that the supplementation of probiotics and prebiotics may play a positive role in stabilizing intestinal immune function and protecting patients with NAFLD/NASH from the effects of increased intestinal permeability (Supplementary Table S1).

In a total of 12 [50,51,52,53,54,55,56,57,58,59,60,61] preclinical studies, researchers employed three types of NGPs: *Akkermansia muciniphila*, *Faecalibacterium prausnitzii*, and *Clostridium butyricum*, along with one prebiotic (Xylo-oligosaccharides, XOS) as treatment modalities. Specifically, *A. muciniphila* was utilized in six studies, five of which exclusively focused on live strain and one employing heat-killed *A. muciniphila*. These investigations were predominantly conducted across Asia (7 studies) and Europe (5 studies). The animal models employed encompassed C57BL/6 mice, Leiden mice, SD rats, and Wistar rats, the majority being male rodents, with only one study involving female animals. Although initial weights of the animals were not reported within these studies, some documented the trend of weight changes before and after treatment administration. In control groups where NGPs were incorporated into the diet in lyophilized form, an HFD was typically adopted as the standard dietary regimen. Conversely, when NGPs were administered via a gavage methodological approach for intervention purposes, phosphate-buffered saline (PBS) served as a common control group medium solution. The dosage regimens varied significantly across these investigations, ranging from 10^7^ CFU/body weight/day to 10^9^ CFU/animal, while intervention durations spanned between 4 to 28 weeks accordingly. Collectively, these findings indicate that NGPs exhibit favorable effects in combating oxidative stress levels while reducing liver inflammation markers and improving liver function indicators while also regulating blood lipid profiles and glucose levels (Supplementary Table S2).

### 3.3. Analysis of Outcomes

#### 3.3.1. Main Findings of Clinical Studies

##### Alanine Aminotransferase (ALT)

We conducted a meta-analysis of 20 studies using a random effects model to evaluate the impact of traditional probiotics on ALT levels in patients with NAFLD/NASH. The calculated combined effect size of −7.87 (95% CI: −11.65 to −4.09) and heterogeneity test (I^2^ = 94%, *p* < 0.01) not only showed a significant therapeutic effect but also highlighted the need for further exploration of heterogeneity among studies (Supplementary Figure S1A). Subgroup analysis by country differences revealed that studies from Iran were statistically different from those in other regions, suggesting a potential role of national factors in the effectiveness of probiotics (Supplementary Table S3). Supplementary Figure S2A presents the network diagram for the network meta-analysis used for AST, while Supplementary Figure S3A shows that “Ref+L+B+Pre” is the only effective probiotic combination (MD: −13.34; 95% CI: −21.13, −5.55). Supplementary Figures S4A and S5A display the corrected funnel plots and network alliance diagrams for the main outcomes, where the upper triangle includes the collective effect sizes of direct comparisons in our network, and the lower triangle contains the estimated effect sizes for each comparison. Additionally, through meta-regression analysis, we identified that baseline ALT, baseline AST, and age could significantly affect post-treatment ALT levels (*p* < 0.05), although high heterogeneity was observed among these factors, their adjusted R-squared (Adj. R^2^%) values suggest that these variables still have a relatively large explanatory power on the trend of changes in post-treatment ALT levels (Table 1, Supplementary Table S4, Supplementary Figures S6A–S9A).

#### 3.3.2. Secondary Findings of Clinical Studies

##### Aspartate Transaminase (AST)

For the analysis targeting AST, we also applied a random effects model, merging data from 16 studies. The results showed an overall effect size of −7.05 (95% CI: −11.60 to −2.51), reflecting the effectiveness of probiotic treatment while also indicating high heterogeneity among the related studies (I^2^ = 72%, *p* < 0.01) (Supplementary Figure S1B). Further network meta-analysis and meta-regression did not find significant differences in the effect of specific probiotic combinations on reducing AST levels, suggesting that the overall effect of probiotics may not depend on specific combinations (Supplementary Figures S2B and S3B). Additionally, the analysis indicated that the country of the study, baseline AST levels, and age might be factors affecting AST levels after traditional probiotic treatment (Table 1, Supplementary Tables S3 and S4, Supplementary Figures S4B–S9B).

##### Gamma Glutamyl Transferase (GGT)

In our meta-analysis investigating the impact of probiotic treatment on GGT levels, we systematically analyzed 10 studies. We found that probiotic treatment significantly reduced GGT levels (overall effect size: −8.02, 95% CI: −14.90 to −1.15) despite very high heterogeneity among the studies (I^2^ = 91%, *p* < 0.01) (Supplementary Figure S1C). Through various statistical analysis methods such as subgroup analysis, network meta-analysis, and meta-regression, we attempted to identify the sources of heterogeneity. However, we were unable to find significant differences across multiple dimensions, including country, probiotic combination, CFU, baseline ALT, baseline AST, and age. This suggests that changes in GGT levels may involve more complex biological mechanisms and necessitate further in-depth research to explore (Supplementary Table S4, Supplementary Figures S2C–S9C).

##### Fasting Blood Glucose (FBG)

The analysis of studies examining FBG levels using a fixed effects model revealed that, despite the majority of studies indicating a decrease in FBG levels following treatment, the overall effect size was −0.05, with a 95% CI of (−2.16, 2.07). The heterogeneity among the studies was not significant (I^2^ = 22%; *p* = 0.23) (Supplementary Figure S1D). This suggests that the impact of probiotics on FBG levels is relatively consistent across different studies, with a limited effect of treatment. The analysis of the specific probiotic combination “Ref+L+B” showed a relatively more significant effect, providing a potential direction for future research (Supplementary Figures S2D and S3D). Furthermore, the analysis indicated that CFU significantly affects post-treatment FBG levels (*p* < 0.05), with no heterogeneity (I^2^ = 0%) (Table 1, Supplementary Table S4, Supplementary Figures S4D–S9D).

##### Total Cholesterol (TC)

We evaluated the impact of traditional probiotics on TC levels in patients with NAFLD/NASH using a random effects model. The analysis indicated that, although most studies showed a decrease in TC levels after treatment, the overall effect size was −7.34, with a 95% CI of (−16.35, 1.66). The high heterogeneity among studies (I^2^ = 76%, *p* < 0.01) prompted us to explore potential reasons further (Supplementary Figure S1E). Notably, a country difference analysis revealed that the results from studies in Iran (MD: −25.56; 95% CI: −43.41 to −7.70) were significantly different from other regions, suggesting that different populations might respond differently to probiotic treatment (Supplementary Table S3). Moreover, we did not find that any specific probiotic combination was more effective than placebo in improving TC levels, indicating that future research needs to explore the types and combinations of probiotics more precisely (Supplementary Figures S2E and S3E). Age, as a factor, with its adjusted explanatory power (R^2^% of 83.09%), suggests that there might be differences in treatment responses among different age groups, thus providing clues for personalized medicine (Table 1, Supplementary Table S4, Supplementary Figures S4E–S9E).

##### Triglycerides (TGs)

In our meta-analysis of the effects on TG levels in patients with NAFLD/NASH, we synthesized the results of related studies using a random effects model. We found that traditional probiotic treatment is significantly associated with a reduction in TG levels (overall effect size: −10.17, 95% CI: −19.88 to −0.45). This result confirms the efficacy of probiotics in improving lipid profiles. However, similar to the analysis on TC, there was significant heterogeneity among studies (I^2^ = 90%, *p* < 0.01) (Supplementary Figure S1F), underscoring the importance of exploring factors leading to this variability. A country difference analysis specifically highlighted significant differences in studies from Iran compared to other regions (MD: −22.46, 95% CI: −26.42 to −18.50), revealing that national factors might be crucial in interpreting study outcomes (Supplementary Table S3). Our analysis did not identify any specific probiotic combination with a superior effect on improving TGs, indicating that future research needs to more finely dissect the mechanisms of action of probiotics (Supplementary Figures S2F and S3F). Through meta-regression analysis, we found that baseline AST and age significantly affect changes in TG levels (*p* < 0.05), highlighting the importance of considering these variables when designing probiotic treatment strategies (Supplementary Table S4, Supplementary Figures S4F–S9F).

##### Body Mass Index (BMI)

Our meta-analysis exploring changes in BMI in patients with NAFLD/NASH revealed a slight decrease in BMI following traditional probiotic treatment (overall effect size: −0.51; 95% CI: −1.01 to −0.00) despite significant heterogeneity among the studies (I^2^ = 92%, *p* < 0.01) (Supplementary Figure S1G). A country difference analysis particularly highlighted the unique effect of studies from Iran (MD: −0.25; 95% CI: −0.44 to −0.07), showing statistically significant differences compared to studies from other countries (Supplementary Table S3). Our network meta-analysis further found significant treatment effects with the “Ref+L+B+Pre” and “Ref+L+B” combinations, suggesting that specific probiotic combinations may play a crucial role in improving BMI (Supplementary Figures S2G and S3G). However, our meta-regression analysis indicated that factors such as CFU, baseline ALT, baseline AST, and age did not significantly affect changes in BMI (Supplementary Table S4, Supplementary Figures S4G–S9G).

##### Fibroscan CAP Score

Our analysis, based on a random effects model, synthesized results from relevant studies and showed a decrease in Fibroscan Controlled Attenuation Parameter (CAP) scores after treatment (overall effect size: −0.87; 95% CI: −1.73 to −0.02), with these studies exhibiting high heterogeneity (I^2^ = 78%, *p* < 0.01) (Supplementary Figure S1H). A country difference analysis revealed significant differences between studies from Iran and those from other countries (MD: −1.51; 95% CI: −1.94 to −1.08) (Supplementary Table S3), highlighting the impact of study location on treatment outcomes. Additionally, combinations “Ref+L+Pre” and “Ref+L+B” were found to have significant treatment effects, suggesting that different probiotic combinations may have varying impacts on improving liver fat accumulation (Supplementary Figures S2H and S3H). Despite this, our meta-regression analysis showed that variables such as CFU, baseline ALT, baseline AST, and age did not affect changes in Fibroscan CAP scores (Supplementary Table S4, Supplementary Figures S4H–S9H).

##### Findings from Preclinical Studies

The presence of *A. muciniphila* leads to a significant reduction in serum levels of ALT, AST, and TG by upregulating the expression of anti-inflammatory factors such as peroxisome proliferator-activated receptor alpha (PPAR-α), peroxisome proliferator-activated receptor gamma (PPAR-γ), and insulin-like growth factor (IGF). This subsequently mitigates liver inflammation and hepatocyte damage. *A. muciniphila* specializes in the degradation of mucin proteins within the gut, converting them into SCFAs and tryptophan metabolites, thereby enhancing intestinal barrier function. Moreover, *A. muciniphila* is associated with the production of diverse antimicrobial peptides in the gut that play a pivotal role in maintaining gut microbiome equilibrium and intestinal immune homeostasis. When combined with quercetin, *A. muciniphila* effectively reduces ALT and total cholesterol levels induced by a HFD while simultaneously improving insulin resistance. Furthermore, this combination results in an elevation of primary bile acids, leading to a substantial increase in total plasma bile acid (BA) concentration.

*F. prausnitzii* significantly enhances the levels of acetate, propionate, and butyrate in the gastrointestinal tract. However, there is variability in butyrate production among different strains of *F. prausnitzii*. Strains LC49 and LB8 of *F. prausnitzii* not only markedly elevated the levels of glutathione peroxidase (GSH-PX) and superoxide dismutase (SOD) but also mitigated malondialdehyde (MDA) production, directly reflecting hepatic oxidative stress status. Supplementation with *F. prausnitzii* reduces TG and TC levels in both liver tissue and serum samples from NASH mice models. It ameliorates hepatic lipid accumulation by modulating the expression of genes associated with lipid metabolism such as CD36, FATP5, PPAR-γ, SREBP-1c, FAS, and LPL; restores impaired intestinal barrier function by enhancing tight junction protein expression including ZO-1 and occludin; and alleviates liver inflammation by reducing tumor necrosis factor-alpha (TNF-α), monocyte chemoattractant protein-1 (MCP-1), and interleukin-6 (IL-6). XOS, a type of non-digestible oligosaccharide composed of xylose units, has been found to significantly increase the abundance of *F. prausnitzii* in the gut microbiota composition studies. XOS mitigates epithelial damage induced by HFD consumption while exacerbating it under low-fat diet conditions. Additionally, XOS enhances liver beta-hydroxyacyl-coenzyme A dehydrogenase (β-HAD) activity, resulting in increased fatty acid metabolism within hepatocytes, leading to reduced fat accumulation.

*C. butyricum B1* (CB B1) has demonstrated efficacy in ameliorating hepatic steatosis and inflammation induced by a HFD in mice. *CB B1* exerts its effects through the upregulation of anti-inflammatory proteins, including Foxp3, interleukin-4 (IL-4), and IL-22, while downregulating pro-inflammatory proteins, such as IFN-γ and IL-17, thereby modulating the gut-liver immune system. The administration of *CB* leads to an increase in butyrate concentration in both cecal contents and the liver, promoting the differentiation of CD4+ T cells into anti-inflammatory subtypes (Th2, Th22, Treg) while inhibiting their differentiation into pro-inflammatory subtypes (Th1, Th17). Clinical studies involving oral administration of rosuvastatin (10 mg) combined with CB capsules (1200 mg daily) have shown significant reductions in levels of total bilirubin (TBIL), direct bilirubin (DBIL), ALT, AST, TC, TGs, free fatty acids (FFA), collagen III peptide (PIIIP), type IV collagen (C-IV), hyaluronic acid (HA), laminin (LN), TNF-α, C-reactive protein (CRP), and IL-6 among patients with NAFLD.

### 3.4. Quality Assessment

A comprehensive quality assessment was conducted for all included clinical studies, taking into consideration the utilization of Intention-to-Treat (ITT) or Per-Protocol (PP) analysis methods. Among the studies that employed ITT analysis, it was observed that one study (12.5%) posed a high risk due to non-compliance with predetermined intervention measures (Supplementary Figure S10A,B). In the case of studies utilizing PP analysis, three studies (16.7%) were rated as high risk. The reasons behind this high-risk rating encompassed: (1) inadequate adherence to intended intervention measures, and (2) lack of clarity in various aspects, such as the randomization process, deviation from intended interventions, and missing data (Supplementary Figure S10C,D). The remaining studies were evaluated with ‘Some concerns’ or deemed ‘Low risk’.

In our analysis of all preclinical trials, we found that studies categorized as low risk constituted 8.3%, those with some concerns accounted for 75%, and high-risk studies made up 16.7% (as illustrated in Supplementary Figure S10E,F). In our analysis of all preclinical studies, we observed that low-risk studies accounted for 8.3%, those with some concerns constituted 75%, and high-risk studies comprised 16.7% (as depicted in Supplementary Figure S10E,F). The primary reason for categorizing studies as high risk was attributed to issues concerning the Concealment of Allocation. Factors contributing to an unclear risk assessment in these studies primarily revolved around inadequate reporting on several key aspects, including insufficient information on animal weight, random sequence allocation, concealment of allocation, random housing, blinding of intervention measures, and randomization in outcome assessment. The studies encompass a range of NGPs, with *A. muciniphila* being the most frequently investigated. The mechanism of action of *A. muciniphila* involves multiple facets. Firstly, it enhances gut microbiota and fortifies intestinal barrier function, thereby reducing levels of ALT and AST and ameliorating liver inflammation. Secondly, *A. muciniphila* regulates bile acid metabolism, thereby modulating TG levels and improving hepatic metabolic function. Additionally, *A. muciniphila* indirectly augments the intestinal antioxidative milieu by promoting SCFA production, thus mitigating oxidative stress and comprehensively improving the symptoms in patients with NAFLD/NASH.

### 3.5. Publication Bias

Given the considerable statistical heterogeneity (>90%) and the high uncertainty in bias risk across most studies, a thorough examination of the evidence base using Egger’s plot indicated the potential presence of publication bias in certain studies (*p* < 0.05).

## 4. Discussion

**Figure 2:**
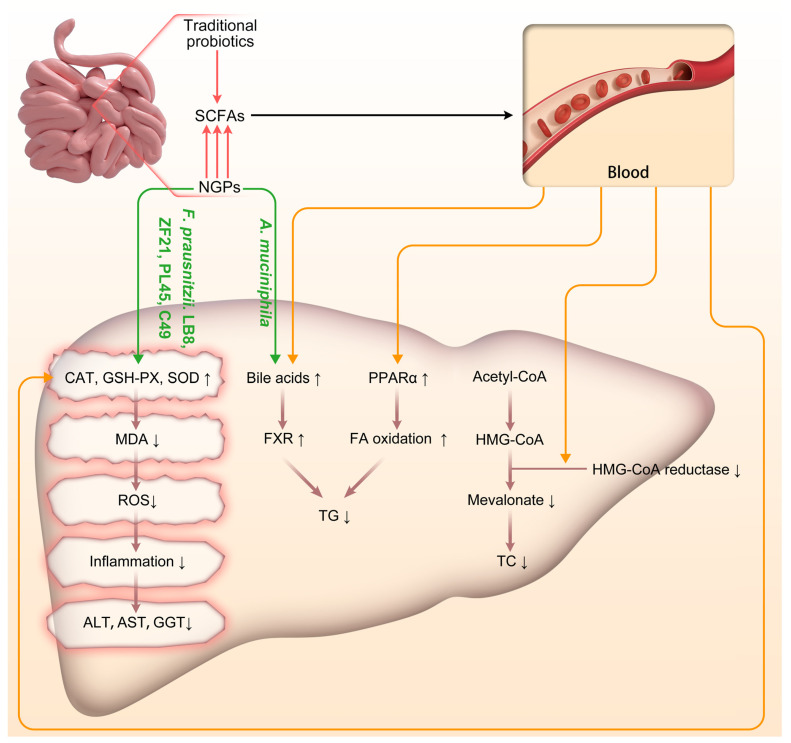
Schematic representation of the impact of traditional probiotics and NGPs on the progression of NAFLD/NASH. Acetyl-CoA: Acetyl-Coenzyme A; *A. muciniphila*: *Akkermansia muciniphila*; CAT: catalase; *F. prausnitzii*: *Faecalibacterium prausnitzii*; FA: fatty acid; FXR: Farnesoid X Receptor; GSH-PX: glutathione peroxidase; HMG-CoA: 3-Hydroxy-3-Methylglutaryl-Coenzyme A; MDA: malondialdehyde; NGPs: next-generation probiotics; PPAR-α: peroxisome proliferation activated receptor α; ROS: reactive oxygen species; SCFAs: short-chain fatty acids; SOD: superoxide dismutase; TC: Total Cholesterol; TG: triglyceride. ↑: up-regulated; ↓: down-regulated.

Early intervention in NAFLD/NASH not only enhances patients’ quality of life but also reduces individual and societal medical costs [62]. The multifaceted mechanisms by which probiotics alleviate NAFLD/NASH include modulation of gut microbiota composition and metabolic activity, suppression of inflammatory cytokine release, improvement of intestinal barrier integrity to mitigate liver inflammation and endotoxin- or inflammatory factor-induced damage, enhancement of insulin sensitivity while reducing hepatic fat accumulation, as well as production of small quantities of beneficial metabolic by-products such as SCFAs that reinforce antioxidative defense mechanisms and diminish oxidative stress [63,64]. Indeed, compared to traditional probiotics in treating NAFLD/NASH, NGPs excel in their ability to enhance the regulation of oxidative stress in the body through diverse pathways [15]. This significant distinction underscores the disparity between NGPs and traditional probiotic therapy for NAFLD/NASH. Despite heterogeneity among the studies included in this review, the findings not only validate the efficacy of traditional probiotics in treating NAFLD/NASH and their impact on associated factors but also delve into the mechanisms by which NGPs modulate oxidative stress responses in NAFLD/NASH (Figure 2).

In this review, the traditional probiotic treatment effects included were all clinical trials. These trials consistently demonstrated the positive therapeutic significance of traditional probiotics in terms of slowing disease progression, reducing systemic and tissue inflammation, and improving insulin resistance. A comprehensive meta-analysis incorporating multiple clinical trials revealed that traditional probiotic treatment significantly lowered levels of ALT, AST, GGT, TG, BMI, and Fibroscan CAP scores. However, the meta-analysis findings revealed a reduction in both FBG and TC indicators following treatment, albeit accompanied by broad confidence intervals for the overall effect size. This suggests an uncertain impact of traditional probiotics on FBG and TC levels. Given the substantial heterogeneity among included studies potentially stemming from variations in inclusion criteria or treatment methods employed across different research settings, network meta-analysis and meta-regression analysis were subsequently conducted to explore factors influencing the efficacy of traditional probiotic treatment. In the network meta-analysis, *Streptococcus thermophilus* and *Lactobacillus bulgaricus* were designated as “Ref” due to their symbiotic relationship and common usage in fermented dairy products. *Bifidobacterium* was classified as “B”, other *Lactobacillus* strains as “L”, prebiotics as “Pre”, and the control group as “Pla”. The network meta-analysis revealed that the combination of “Ref+L+B+Pre” demonstrated efficacy in reducing ALT and BMI levels, while the combination of “Ref+L+B” showed effectiveness in lowering FBG levels and BMI. Moreover, both combinations of “Ref+L+B” and “Ref+L+Pre” exhibited positive effects on improving Fibroscan CAP score. These findings suggest that distinct traditional probiotic combinations may be necessary to achieve optimal therapeutic outcomes for different indicators among NAFLD/NASH patients. According to the results of the meta-regression analysis, age significantly influenced post-treatment levels of ALT, TC, and TG. Additionally, baseline AST levels had a significant impact on post-treatment ALT and TG levels. Furthermore, only baseline ALT levels exhibited a significant influence on post-treatment ALT levels, while CFU solely affected post-treatment FBG levels. These findings suggest that patient age and liver inflammation markers, such as baseline ALT and AST levels, play a crucial role in determining the efficacy of traditional probiotics for treating NAFLD/NASH. Therefore, treatment strategies for NAFLD/NASH patients should not only focus on increasing CFU but also consider adjusting the combination of traditional probiotics based on individual age and liver health status. Such personalized approaches may lead to more effective improvements in treatment. Therefore, in the treatment of patients with NAFLD/NASH, it is imperative to not only consider augmenting the CFU but also to meticulously tailor the combination of conventional probiotics based on the patient’s age and liver health status. Such a personalized therapeutic approach holds promise for enhancing treatment efficacy.

Subgroup analysis results revealed that regional factors exerted a significant influence on the efficacy of conventional probiotics in managing NAFLD/NASH patients, particularly with regards to ALT, AST, TC, TG, BMI, and Fibroscan CAP score indicators. Notably, the findings from Iran exhibited noteworthy variations in these indicators compared to other countries. This divergence may be attributed to distinct characteristics, dietary habits, lifestyles, medical practices, or genetic backgrounds specific to the Iranian population when contrasted with other nations. However, due to insufficient elaboration on these factors across all included studies, further meta-regression analysis was not conducted to explore such disparities.

Despite the potential benefits of NGPs in treating NAFLD/NASH, most studies remain in the preclinical phase, with only a few progressing to clinical research. This may be attributed to challenges faced in human studies, including inadequate preclinical support for specific NGP treatment methods such as oral administration or fecal microbiota transplantation. Furthermore, comprehensive evaluation of NGP efficacy requires long-term follow-up experimental designs akin to prospective studies that are often associated with higher costs. The research includes a variety of NGPs, with *A. muciniphila* being one of the most used. The mechanism of action of *A. muciniphila* in treating NAFLD/NASH is multifaceted [52,53,54,57,58,59,60]. Firstly, *A. muciniphila* alters the composition of the gut microbiota and enhances intestinal barrier function, thereby reducing liver damage by decreasing the transintestinal permeation of inflammatory mediators and harmful substances, which in turn lowers ALT and AST levels. Secondly, *A. muciniphila* modulates bile acid metabolism, thereby adjusting TG levels and improving liver metabolic function. Thirdly, *A. muciniphila* produces more SCFAs compared to traditional probiotics, especially butyrate, which regulates the expression of antioxidative enzymes in liver cells. Thus, *A. muciniphila* indirectly enhances the antioxidative capacity of liver cells, helping to alleviate oxidative stress and comprehensively improve the symptoms of patients with NAFLD/NASH. Finally, *A. muciniphila* plays a role in regulating immune and inflammatory responses. It modulates inflammation by regulating γδT17 cells activated by Toll-Like Receptor 2 (TLR2) and may influence the transition of macrophages from a pro-inflammatory (M1) to an anti-inflammatory (M2) state. These shifts help to reduce inflammation and prevent the progression of NASH. In the analysis, we uncovered some interesting findings. Firstly, heat-inactivated *A. muciniphila* was able to reduce the hypertrophy of adipocytes in mesenteric white adipose tissue induced by HFDs. However, it did not significantly impact overall obesity or the storage weight of white adipose tissue. Secondly, heat-inactivated *A. muciniphila* did not influence the progression of liver inflammation and fibrosis. Furthermore, heat-inactivated *A. muciniphila* decreased the production of butyrate and valerate within the gastrointestinal tract. These findings suggest that the therapeutic effects of heat-inactivated *A. muciniphila* on NAFLD/NASH are quite limited. The study [55] of specific strains of *F. prausnitzii*, namely A2-165, LB8, ZF21, PL45, and LC49, exhibited significant improvements in serum lipid profiles and reductions in glucose intolerance, adipose tissue dysfunction, hepatic steatosis, inflammation, and oxidative stress. Notably, strains LB8 and LC49 demonstrated a substantial increase in SCFA production, thereby enhancing the indirect antioxidant capabilities of *F. prausnitzii*. Moreover, strains LB8, ZF21, PL45 and LC49 also directly augmented the activity of antioxidative enzymes such as GSH-PX and SOD within the liver while reducing MDA production. This effectively alleviated oxidative stress in hepatic cells. The direct antioxidative action by strains LB8, ZF21, PL45, and LC49 represents a promising approach for mitigating oxidative stress associated with NAFLD/NASH, highlighting its potential for managing these conditions. According to research by Shin et al. [56], *F. prausnitzii* effectively regulates key genes involved in lipid metabolism, including CD36, Fatty Acid Transport Protein 5 (FATP5), PPAR-γ, Sterol Regulatory Element-Binding Protein-1c (SREBP-1c), Fatty Acid Synthase (FAS), and Lipoprotein Lipase (LPL). By influencing these transport proteins and enzymes associated with lipid synthesis, *F. prausnitzii* effectively mitigates hepatic steatosis. Furthermore, specific strains of *F. prausnitzii* (EB-FPDK9, EB-FPDK11, and EB-FPYYK1) can reduce the expression of Toll-Like Receptor 4 (TLR4), thereby lowering levels of pro-inflammatory cytokines (TNF-α, MCP-1, IL-6) in mice with NASH, subsequently improving inflammatory responses in NASH. The study also reveals an interesting phenomenon: elevated levels of SCFAs can perpetuate inflammatory conditions in the liver or cecum. However, *F. prausnitzii* can alleviate these inflammatory states by reducing SCFAs to normal levels. This effect may be related to the overactivation of the immune system due to excessively high levels of SCFAs. Overall, *F. prausnitzii* has demonstrated significant efficacy in ameliorating symptoms associated with NAFLD/NASH induced by HFD. Its specific strains not only augment hepatic antioxidative enzyme activity but also mitigate liver oxidative stress through inflammation reduction, thereby opening new avenues for future research on NAFLD/NASH treatment through the regulation of oxidative stress responses mediated by NGPs. *C. butyricum* and its byproduct, butyrate, exert significant therapeutic effects in the treatment of NAFLD/NASH. These therapeutic effects primarily stem from the ability of butyrate to enhance intestinal barrier function, reduce the translocation of endotoxins that can induce hepatic oxidative stress, and regulate gut microbiota to maintain a balance between oxidation and antioxidation, thereby modulating immune responses, suppressing inflammation, and improving hepatic metabolic function. Furthermore, clinical studies have demonstrated that combining *C. butyricum* capsules with statins significantly enhances treatment efficacy while reducing the progression of liver fibrosis [50,51]. The collective findings from preclinical and clinical investigations underscore the substantial potential of *C. butyricum* and its associated production of butyrate in NAFLD/NASH therapy [49]. According to Yang et al. [51], a dosage of 5 × 10^7^ CFU of *C. butyricum* is sufficient to promote the production of SCFAs, thereby improving liver inflammation and lipid accumulation. Similarly, Shin et al. [56] suggested that a higher dosage of 10^8 CFU of *F. prausnitzii* is necessary to effectively stimulate SCFA production for regulating immune responses, maintaining oxidative and antioxidative balance, and ultimately ameliorating liver inflammation and lipid accumulation. Recommending specific doses of NGPs is therefore deemed impractical.

Fructooligosaccharides (FOS) are widely recognized as a prevalent type of prebiotics, renowned for their ability to stimulate the growth and activity of beneficial gut bacteria, particularly *Bifidobacterium* and *Lactobacillus* [65]. Upon fermentation, FOS yields modest quantities of SCFAs, predominantly acetate, propionate, and butyrate. These SCFAs effectively augment mucus production and facilitate the proliferation of healthy cells, thereby fortifying the integrity of the intestinal barrier. XOSs are a type of non-digestible prebiotic that remain intact in the upper gastrointestinal tract and reach the colon, where they undergo fermentation by the gut microbiota. XOS has been demonstrated to enhance the abundance of *F. prausnitzii* in the colon significantly [61]. In conditions of HFD, XOS can mitigate epithelial damage caused by such diets; however, under low-fat-diet conditions, XOS may exacerbate this damage. Furthermore, XOS augments the activity of liver beta-hydroxyacyl-CoA dehydrogenase (β-HAD), thereby promoting fatty acid metabolism in hepatic cells and reducing fat accumulation. The differential effects of XOS under varying dietary backgrounds (high-fat versus low-fat) indicate that the action of XOS can be significantly influenced by dietary factors. This observation highlights a crucial avenue for future research to investigate the mechanisms and impacts of prebiotics in diverse dietary conditions. Understanding these interactions is pivotal for developing more efficacious dietary interventions and prebiotic therapies targeting gut health and associated conditions, such as NAFLD/NASH.

NGPs can modulate the liver’s antioxidative activity through both direct and indirect mechanisms, involving intricate interactions among the gut microbiota, the gut-liver axis, and various metabolic and immune pathways. In terms of direct antioxidative action, our findings demonstrate that specific strains of *F. prausnitzii*, namely LB8, ZF21, PL45, and LC49 can upregulate hepatic expression of key antioxidative enzymes such as GSH-PX and SOD. These enzymes augment the liver’s capacity to counteract ROS. Additionally, *F. prausnitzii* LB8, ZF21, PL45, and LC49 effectively mitigate levels of MDA (an oxidative stress marker of lipids) in the liver, thereby safeguarding hepatocytes against oxidative damage (Figure 2). The indirect antioxidative actions of NGPs are primarily mediated by SCFAs, particularly butyrate. Butyrate exerts its antioxidative effects through several crucial mechanisms. Firstly, it helps maintain the integrity of the intestinal barrier, reducing endotoxin entry into the bloodstream and thereby enhancing the body’s antioxidative defenses via the gut-liver axis [66]. Secondly, butyrate activates the Nrf2 pathway, promoting the expression of antioxidative enzymes and alleviating oxidative stress in liver cells [67]. Additionally, butyrate effectively inhibits oxidative DNA damage. Acetate and propionate not only impact glucose production in the liver, potentially improving glucose homeostasis and insulin sensitivity, but also regulate lipid metabolism in the liver by reducing fatty acid and cholesterol synthesis, thus mitigating hepatic steatosis [68]. Our comparison further reveals that NGPs have a greater potential for direct and efficient production of specific SCFAs compared to traditional probiotics; specifically, *A. muciniphila* and *C. butyricum* exhibit stronger capabilities in producing butyrate. The indirect antioxidative effects of NGPs are also achieved through the regulation of bile acid metabolism, which activates nuclear receptors and signaling pathways in the liver (such as FXR and TGR5) that influence oxidative stress responses. Our included studies have shown that *A. muciniphila* affects the composition and circulation of bile acids in the intestine, indirectly regulating liver function and the gut-liver axis to respond to oxidative stress.

The distinguishing characteristics of network meta-analyses and systematic reviews lie in their extensive evidence base, enabling the evaluation of multiple studies while accounting for inter-study variability. This article delves into the role of traditional probiotics and NGPs in NAFLD/NASH. The selection of literature followed widely recommended and approved systematic review practices to ensure comprehensive coverage and depth of analysis. Moreover, we employed ROB 2 tools recommended by Cochrane and SYRCLE’s RoB to evaluate publication bias in preclinical and clinical studies, respectively. Our bias risk analysis revealed certain deficiencies in experimental design across some studies, underscoring the need for improved reporting guidelines and experimental design standards for animal experiments to enhance the quality and reliability of scientific evidence.

Notably, considerable heterogeneity was observed across all assessment parameters, indicating variations in measurement outcomes among different studies. Importantly, most studies failed to specify the applicability of their findings to other species, including humans. Considering the experimental models employed in most studies and the global significance of NAFLD/NASH, it is imperative to translate research discoveries on NGPs into precise treatments for NAFLD/NASH.

Based on our research findings, we propose a comprehensive set of recommendations for the treatment of NAFLD/NASH with NGPs. Firstly, we recommend systematic studies on the mechanisms by which NGPs alleviate oxidative stress and promote lipid metabolism improvement, including the clarification of their direct and indirect roles in the antioxidant process. This step lays a theoretical foundation for basic research and subsequent clinical applications. Secondly, given that current studies are mostly based on animal models, we emphasize the importance of conducting extensive human clinical trials to validate the actual effects and safety of NGPs in treating NAFLD/NASH. This would not only enhance the reliability of the research but also provide direct evidence for clinical application. Furthermore, considering that traditional probiotics and NGPs may have different mechanisms of action and therapeutic effects, we suggest exploring the possibility of combining them in treatment. This integrated therapeutic approach could potentially create a synergistic effect, improving treatment outcomes. Lastly, recognizing the differences in pathological characteristics and gut microbiome compositions among patients, we advise future research to focus on developing personalized NGP treatment plans based on specific pathological features and gut microbiome compositions of patients. This approach aims to tailor treatments more effectively to individual patient needs, potentially enhancing the efficacy of NGPs in managing NAFLD/NASH.

## 5. Limitations

This study’s limitations include the heterogeneity among the included research, potentially stemming from differences in measurement outcomes across various studies. These differences have weakened the consistency of our analysis results and limited their broad applicability. To explore and understand the roots of these discrepancies, we employed statistical techniques such as meta-regression analysis and subgroup analysis. Through these methods, we have recognized that future research should strive to adopt uniform measurement units and reporting standards to reduce such heterogeneity, thereby enhancing the accuracy of meta-analytical results. Although NGPs have shown significant potential in the treatment of NAFLD/NASH, most studies remain in the preclinical stage, underscoring the urgent need for clinical trials to validate the therapeutic effects and safety of NGPs.

It is particularly noteworthy that lifestyle changes remain the major management for NAFLD/NASH. These modifications primarily involve dietary adjustments, increasing physical activity, weight loss, and avoiding the intake of alcohol and other substances harmful to the liver [69]. Dietary recommendations include reducing the intake of saturated fats, sugars, and refined carbohydrates, and increasing the proportion of dietary fiber, vegetables, and fruits [70]. Regular physical activity, especially aerobic exercises and strength training, can improve muscle insulin sensitivity and promote fat accumulation, thereby helping to decrease liver fat content [70]. Weight management is particularly crucial for overweight or obese patients with NAFLD/NASH [71]. On this foundation, probiotics as supplements can have a positive impact on the balance of gut microbiota, thereby intervening in the pathophysiological process of NAFLD/NASH from multiple dimensions. Probiotics not only directly affect the liver’s metabolic pathways, improving insulin sensitivity and reducing liver fat accumulation, but can also indirectly lower oxidative stress levels and systemic inflammatory responses by improving gut health [3,4]. Nevertheless, the assessing of the combined impact of lifestyle modification and probiotics supplementation on NAFLD/NASH management is beyond the scope of this review. Therefore, we strongly recommend that future studies focus on thoroughly assessing the combined impact of probiotics with different lifestyle change measures, such as dietary adjustments, increased physical activity, and weight loss strategies, on the treatment outcomes of NAFLD/NASH to enrich the current evidence base and provide clear guidance for clinical practice.

## 6. Conclusions

Our objective was to provide a comprehensive overview of the existing literature regarding the utilization of traditional probiotics and NGPs for the treatment of NAFLD/NASH based on disease-specific characteristics. Meta-analyses investigating the effects of traditional probiotics in NAFLD/NASH patients have demonstrated favorable therapeutic outcomes, encompassing attenuation of inflammatory mediators, modulation of lipid metabolism, amelioration of hepatic fibrosis, facilitation of weight management, and obesity control. Meta-regression and subgroup analyses have identified pivotal factors influencing treatment response, including probiotic strain composition, patient age, baseline ALT and AST levels, as well as nationality. Intriguingly, CFU solely exhibited a significant impact on glycemic regulation. In the management of NAFLD/NASH, early use of traditional probiotics, selective strains, and reduction of liver inflammation can improve treatment outcomes and align with personalized medicine principles. NGPs not only offer similar therapeutic effects as traditional probiotics but also directly modulate gene expression in liver antioxidative pathways and reduce oxidative stress markers in the liver. Moreover, NGPs indirectly regulate oxidative stress responses by increasing butyrate content in intestinal SCFAs. In this context, *A. muciniphila* and *C. butyricum* demonstrate a stronger ability to produce butyrate. In summary, although *A. muciniphila* is extensively studied as an NGP, the findings of this review indicate that its antioxidative stress capabilities are comparatively less pronounced than those of *F. prausnitzii* and *C. butyricum*, particularly in terms of direct antioxidative stress responses where it exhibits slightly lower effectiveness than *F. prausnitzii* LC49. Despite the superior performance of NGPs in combating oxidative stress compared to traditional probiotics, there is currently no consensus in the literature regarding the optimal fermentable substrates, dosages, and treatment durations for NGPs. Further experimental research is warranted to investigate these parameters. Considering that NGPs are already being utilized in clinical settings, but evidence of their potential adverse reactions (beyond known contraindications) remains insufficient, future research should prioritize conducting additional clinical trials to ensure their safety and efficacy.

## Figures and Tables

**Table 1:** Summary of factors significantly influencing the therapeutic effects of probiotics on clinical parameters related to NAFLD/NASH as assessed in this review.

Clinical Parameters	Influencing Factors	Heterogeneity		Adjusted R-Squared (Adj. R^2^%)
		I^2^	*p*	
Alanine aminotransferase (ALT)	baseline ALT	88.96	<0.05	25.54
	baseline AST	86.91	<0.05	36.55
	Age	88.02	<0.05	29.31
Aspartate transaminase (AST)	baseline AST	60.80	>0.05	21.47
	Age	59.53	>0.05	21.92
Fasting blood glucose (FBG)	CFU	0	<0.05	100
Total cholesterol (TC)	Age	35.90	<0.05	83.09
Triglycerides (TGs)	baseline AST	50.50	<0.05	73.36
	Age	50.81	<0.05	57.11

## Data Availability

Data available upon request.

## Supplementary Materials

The following supporting information can be downloaded at: https://www.mdpi.com/article/10.3390/antiox13030329/s1, Supplementary Table S1: Baseline characteristics of the included clinical studies; Supplementary Table S2: Baseline characteristics of the included preclinical studies; Supplementary Table S3: Subgroup analysis; Supplementary Table S4: Meta-regression output; Supplementary Figure S1: Meta-analysis of pooled clinical studies; Supplementary Figure S2: Net work plot of different traditional probiotics either alone or combination; Supplementary Figure S3: Meta-analysis of different traditional probiotics either alone or combination; Supplementary Figure S4: Comparison-adjusted funnel plot of different probiotics comparison; Supplementary Figure S5: Network alliance plot of different probiotics; Supplementary Figure S6: Meta-regression analysis of CFU and mean difference; Supplementary Figure S7: Meta-regression analysis of the effect of ALT baseline; Supplementary Figure S8: Meta-regression analysis of the effect of AST baseline; Supplementary Figure S9: Meta-regression analysis of the effect of Age; Supplementary Figure S10: Risk of bias analysis was performed on the 38 included research studies with the ROB2 tool and SYRCLE ROB tool.
