# Supplementary material for: Roles of Traditional and Next-Generation Probiotics on Non-Alcoholic Fatty Liver Disease (NAFLD) and Non-Alcoholic Steatohepatitis (NASH): A Systematic Review and Network Meta-Analysis
Source: Antioxidants (Basel). 2024 Mar 7;13(3):329. doi: 10.3390/antiox13030329 (PMC10968178; doi:10.3390/antiox13030329)
Supplement: Supplementary file 1 [file antioxidants-13-00329-s001.zip › Supplementary figure collection 09012024.pdf]

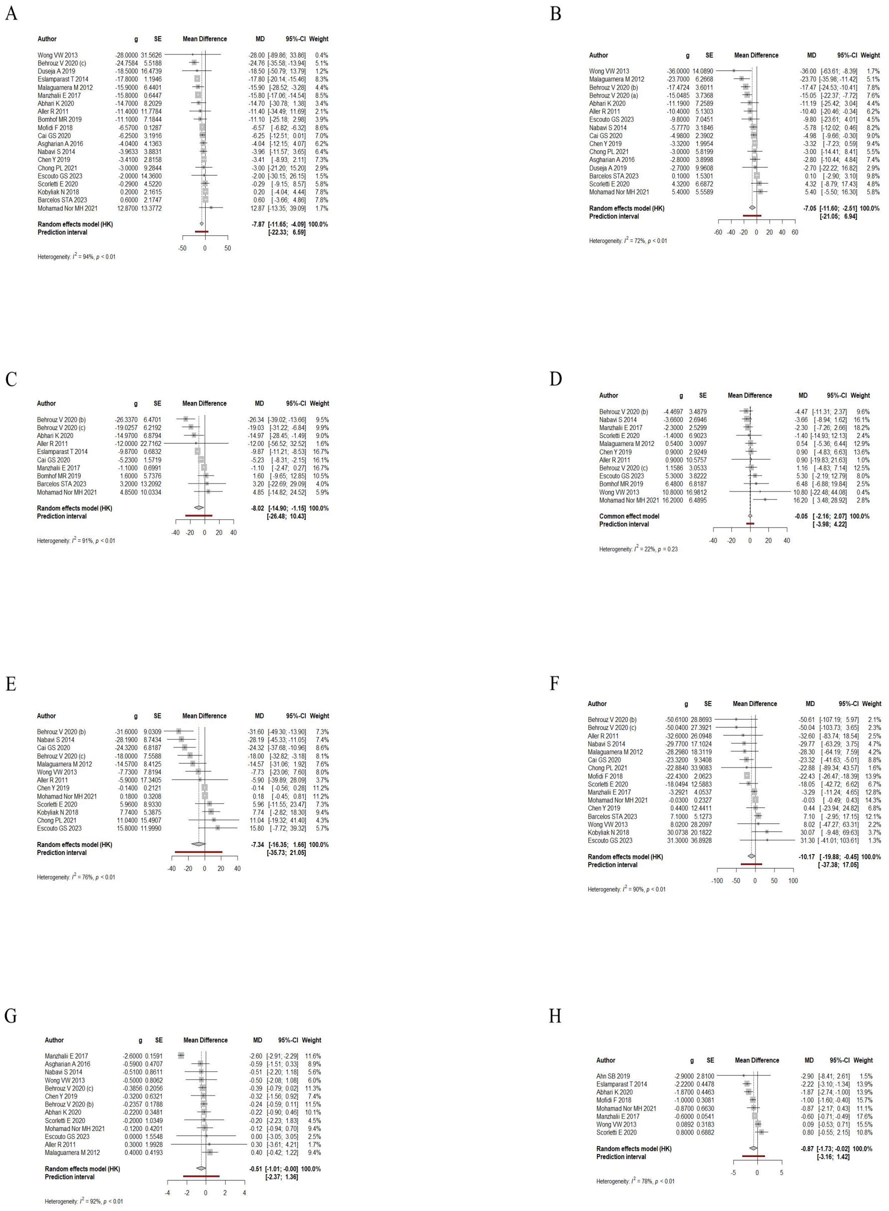

Supplementary Figure S1. Meta-analysis of pooled clinical studies for (A) alanine aminotransferase (ALT), (B) aspartate transaminase (AST), (C) gamma glutamyl transferase (GGT), (D) fasting blood glucose (FBG), (E) total cholesterol (TC), (F) triglycerides (TG), (G) body Mass Index (BMI), and (H) fibroscan CAP score. Forest plot is represented by mean with 95% confidence interval.

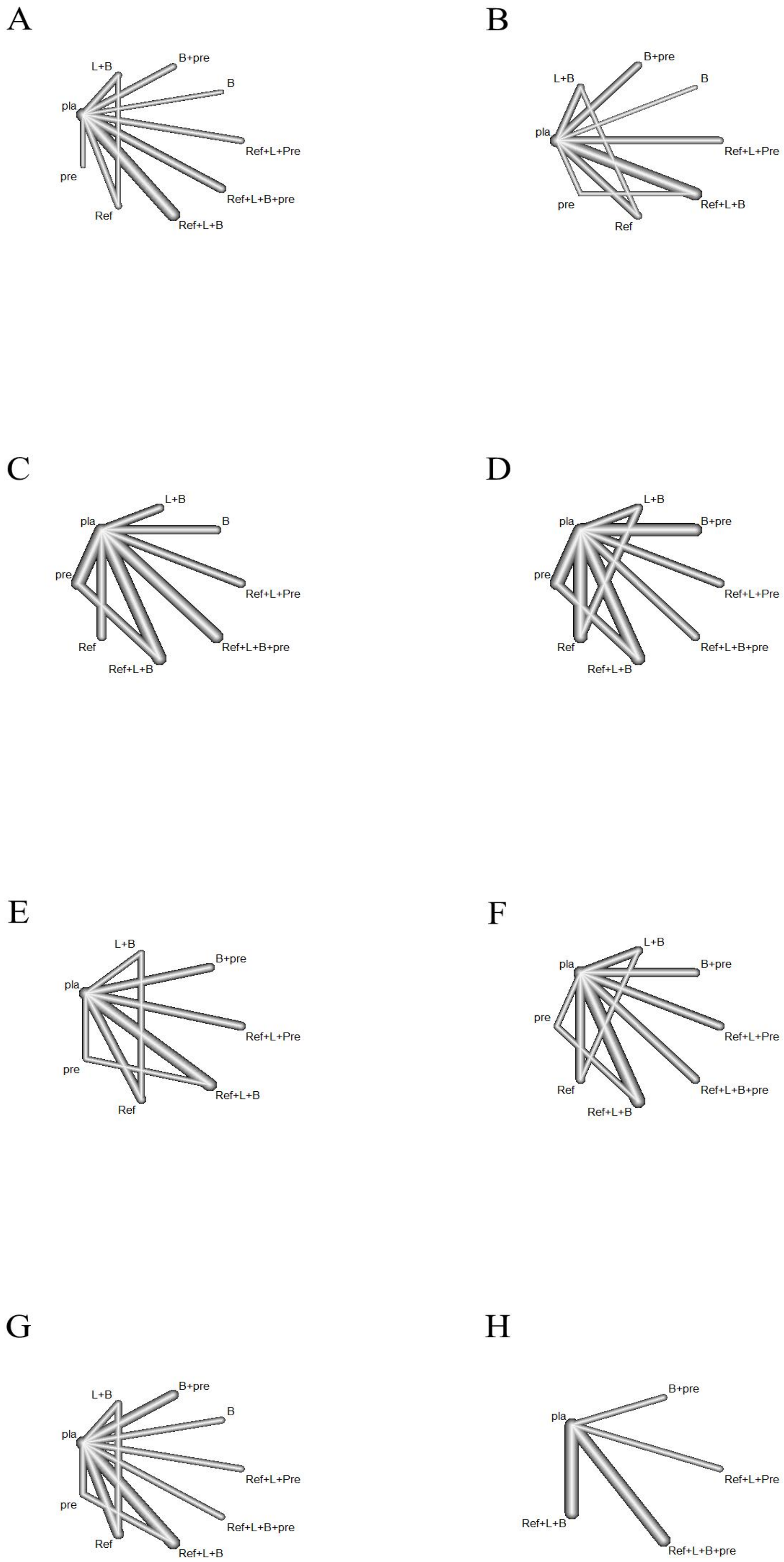

Supplementary Figure S2. Network plot of different traditional probiotics either alone or combination for (A) alanine aminotransferase (ALT), (B) aspartate transaminase (AST), (C) gamma glutamyl transferase (GGT), (D) fasting blood glucose (FBG), (E) total cholesterol (TC), (F) triglycerides (TG), (G) body Mass Index (BMI) and (H) fibroscan CAP score.Thicker lines typically signify that there are more studies comparing the two treatments, which also indicates a higher precision in assessing the effect size between the two therapies. B: *Bifidobacterium*; L: *Lactobacillus;* Pla: *control group;* Pre: *prebiotics*; Ref: *Streptococcus thermophilus* and *Lactobacillus bulgaricus*.

A

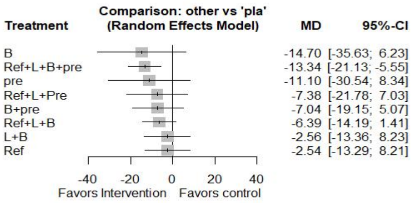

B

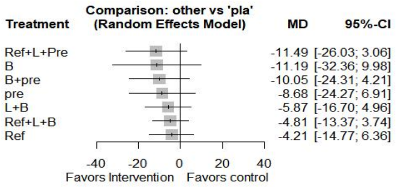

C

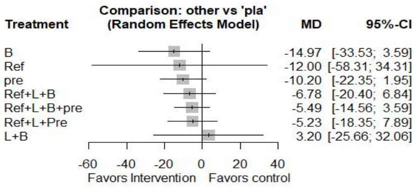

D

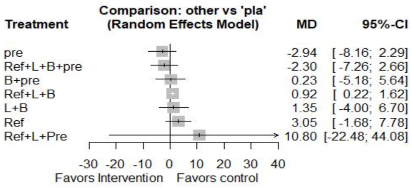

E

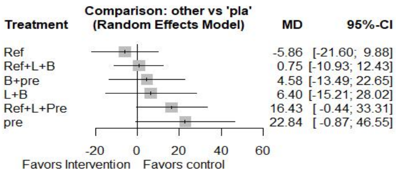

F

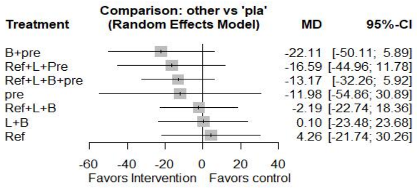

G

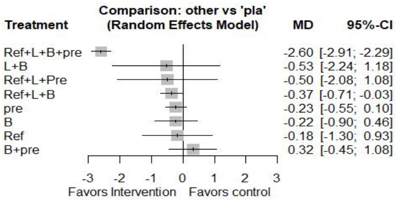

H

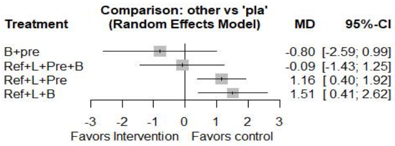

Supplementary Figure S3. Meta-analysis of different traditional probiotics either alone or combination for (A) alanine aminotransferase (ALT), (B) aspartate transaminase (AST), (C) gamma glutamyl transferase (GGT), (D) fasting blood glucose (FBG), (E) total cholesterol (TC), (F) triglycerides (TG), (G) body Mass Index (BMI) and (H) fibroscan CAP score. B: *Bifidobacterium*; L: *Lactobacillus*; Pla: *control group*; Pre: *prebiotics*; Ref: *Streptococcus thermophilus* and *Lactobacillus bulgaricus*.

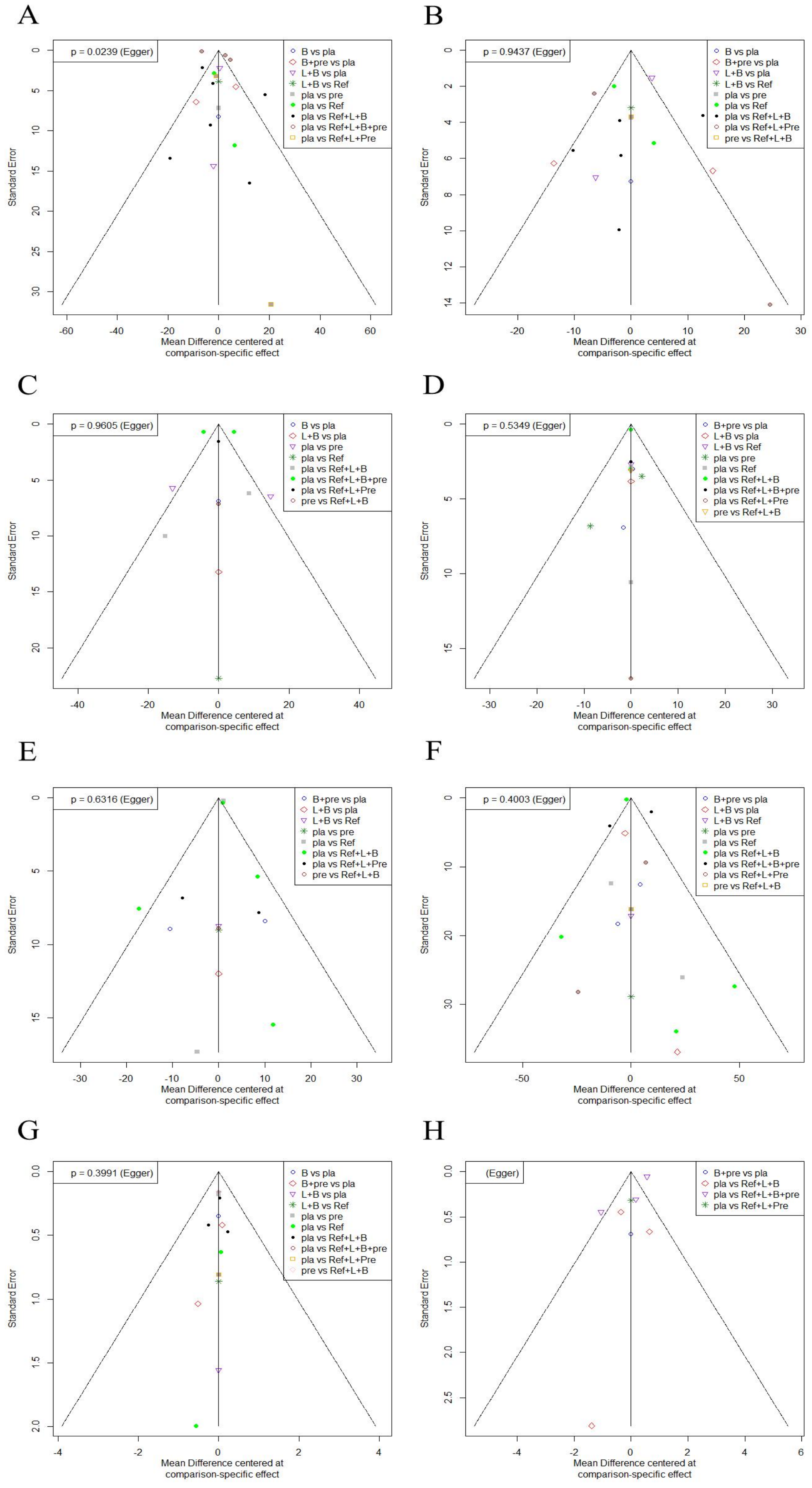

Supplementary Figure S4. Comparison-adjusted funnel plot of different probiotics comparison for (A) alanine aminotransferase (ALT), (B) aspartate transaminase (AST), (C) gamma glutamyl transferase (GGT), (D) fasting blood glucose (FBG), (E) total cholesterol (TC), (F) triglycerides (TG), (G) body Mass Index (BMI) and (H) fibroscan CAP score. A dot closer to the center line (at mean difference = 0) suggests that the study's effect size is close to the overall meta-analysis effect, implying agreement with the meta-analytic mean. B: *Bifidobacterium*; L: *Lactobacillus*; Pla: *control group*; Pre: *prebiotics*; Ref: *Streptococcus thermophilus* and *Lactobacillus bulgaricus*.

A

B	.	.	-14.70 (-35.63; 6.23)	.	.	.	.	.
-7.66 (-31.84; 16.52)	B+pre	.	-7.04 (-19.15; 5.07)	.	.	.	.	.
-12.14 (-35.69; 11.42)	-4.47 (-20.70; 11.75)	L+B	0.16 (-12.66; 12.98)	.	-3.96 (-19.38; 11.45)	.	.	.
-14.70 (-35.63; 6.23)	-7.04 (-19.15; 5.07)	-2.56 (-13.36; 8.23)	Pla	11.10 (-8.34; 30.54)	5.23 (-7.51; 17.97)	6.39 (-1.41; 14.19)	13.34 (5.55; 21.13)	7.38 (-7.03; 21.78)
-3.60 (-32.17; 24.97)	4.06 (-18.84; 26.97)	8.54 (-13.70; 30.77)	11.10 (-8.34; 30.54)	Pre	.	.	.	.
-12.16 (-35.69; 11.37)	-4.50 (-20.69; 11.69)	-0.02 (-11.75; 11.71)	2.54 (-8.21; 13.29)	-8.56 (-30.78; 13.66)	Ref	.	.	.
-8.31 (-30.65; 14.03)	-0.65 (-15.05; 13.76)	3.83 (-9.49; 17.15)	6.39 (-1.41; 14.19)	-4.71 (-25.66; 16.24)	3.85 (-9.43; 17.14)	Ref+L+B	.	.
-1.36 (-23.70; 20.98)	6.30 (-8.10; 20.70)	10.77 (-2.54; 24.09)	**13.34 (5.55; 21.13)**	2.24 (-18.71; 23.19)	10.80 (-2.48; 24.08)	6.95 (-4.08; 17.97)	Ref+L+B+pre	.
-7.32 (-32.74; 18.09)	0.34 (-18.48; 19.16)	4.81 (-13.19; 22.81)	7.38 (-7.03; 21.78)	-3.72 (-27.92; 20.48)	4.84 (-13.14; 22.81)	0.98 (-15.40; 17.37)	-5.96 (-22.34; 10.42)	Ref+L+Pre
The upper triangle contains the pooled effect sizes of the direct comparisons available in our network. The lower triangle of the matrix contains the estimated effect sizes for each comparison. Significant results are in bold.								

B

B	.	.	-11.19 (-32.36; 9.98)	.	.	.	.	
-1.14 (-26.67; 24.39)	B+pre	.	-10.05 (-24.31; 4.21)	.	.	.	.	
-5.32 (-29.10; 18.46)	-4.18 (-22.09; 13.73)	L+B	-3.55 (-16.23; 9.13)	.	-5.78 (-22.65; 11.10)	.	.	
-11.19 (-32.36; 9.98)	-10.05 (-24.31; 4.21)	-5.87 (-16.70; 4.96)	Pla	15.05 (-2.25; 32.35)	6.36 (-5.84; 18.56)	4.81 (-3.74; 13.37)	11.49 (-3.06; 26.03)	
-2.51 (-28.80; 23.78)	-1.37 (-22.50; 19.76)	2.81 (-16.18; 21.79)	8.68 (-6.91; 24.27)	Pre	.	2.42 (-14.82; 19.67)	.	
-6.98 (-30.64; 16.68)	-5.84 (-23.59; 11.91)	-1.66 (-13.84; 10.52)	4.21 (-6.36; 14.77)	-4.47 (-23.30; 14.36)	Ref	.	.	
-6.38 (-29.21; 16.45)	-5.24 (-21.87; 11.39)	-1.06 (-14.86; 12.74)	4.81 (-3.74; 13.37)	-3.87 (-19.44; 11.71)	0.60 (-12.99; 14.20)	Ref+L+B	.	
0.30 (-25.39; 25.98)	1.44 (-18.93; 21.81)	5.62 (-12.52; 23.75)	11.49 (-3.06; 26.03)	2.81 (-18.51; 24.13)	7.28 (-10.70; 25.26)	6.67 (-10.20; 23.55)	Ref+L+Pre	
The upper triangle contains the pooled effect sizes of the direct comparisons available in our network. The lower triangle of the matrix contains the estimated effect sizes for each comparison. Significant results are in bold.								

C

B	.	-14.97 (-33.53; 3.59)	.	.	.	.	.	
-18.17 (-52.49; 16.15)	L+B	3.20 (-25.66; 32.06)	.	.	.	.	.	
-14.97 (-33.53; 3.59)	3.20 (-25.66; 32.06)	Pla	11.59 (-0.77; 23.94)	12.00 (-34.31; 58.31)	10.39 (-3.71; 24.49)	5.49 (-3.59; 14.56)	5.23 (-7.89; 18.35)	
-4.77 (-26.95; 17.42)	13.40 (-17.91; 44.72)	10.20 (-1.95; 22.35)	Pre	.	-7.31 (-26.23; 11.61)	.	.	
-2.97 (-52.87; 46.93)	15.20 (-39.37; 69.77)	12.00 (-34.31; 58.31)	1.80 (-46.08; 49.68)	Ref	.	.	.	
-8.19 (-31.21; 14.84)	9.98 (-21.93; 41.90)	6.78 (-6.84; 20.40)	-3.42 (-19.12; 12.28)	-5.22 (-53.49; 43.06)	Ref+L+B	.	.	
-9.48 (-30.14; 11.18)	8.69 (-21.57; 38.94)	5.49 (-3.59; 14.56)	-4.72 (-19.88; 10.45)	-6.51 (-53.71; 40.68)	-1.30 (-17.66; 15.07)	Ref+L+B+pre	.	
-9.74 (-32.47; 12.99)	8.43 (-23.28; 40.14)	5.23 (-7.89; 18.35)	-4.97 (-22.85; 12.91)	-6.77 (-54.91; 41.37)	-1.55 (-20.47; 17.36)	-0.26 (-16.21; 15.70)	Ref+L+Pre	
The upper triangle contains the pooled effect sizes of the direct comparisons available in our network. The lower triangle of the matrix contains the estimated effect sizes for each comparison. Significant results are in bold.								

D

B+pre	.	0.23 (-5.18; 5.64)	.	.	.	.	.	
-1.12 (-8.73; 6.49)	L+B	5.30 (-2.19; 12.79)	.	-3.66 (-8.94; 1.62)	.	.	.	
0.23 (-5.18; 5.64)	1.35 (-4.00; 6.70)	Pla	2.20 (-3.88; 8.28)	-0.90 (-6.43; 4.63)	-0.90 (-1.61; -0.20)	2.30 (-2.66; 7.26)	-10.80 (-44.08; 22.48)	
3.17 (-4.35; 10.68)	4.29 (-3.19; 11.76)	2.94 (-2.29; 8.16)	Pre	.	-5.63 (-11.68; 0.42)	.	.	
-2.82 (-10.00; 4.37)	-1.70 (-6.29; 2.90)	-3.05 (-7.78; 1.68)	-5.98 (-13.03; 1.06)	Ref	.	.	.	
-0.69 (-6.14; 4.76)	0.43 (-4.97; 5.83)	**-0.92 (-1.62; -0.22)**	-3.86 (-9.07; 1.36)	2.13 (-2.66; 6.91)	Ref+L+B	.	.	
2.53 (-4.81; 9.87)	3.65 (-3.64; 10.95)	2.30 (-2.66; 7.26)	-0.64 (-7.84; 6.56)	5.35 (-1.51; 12.20)	3.22 (-1.79; 8.23)	Ref+L+B+pre	.	
-10.57 (-44.29; 23.15)	-9.45 (-43.16; 24.26)	-10.80 (-44.08; 22.48)	-13.74 (-47.43; 19.95)	-7.75 (-41.37; 25.87)	-9.88 (-43.17; 23.41)	-13.10 (-46.75; 20.55)	Ref+L+Pre	
The upper triangle contains the pooled effect sizes of the direct comparisons available in our network. The lower triangle of the matrix contains the estimated effect sizes for each comparison. Significant results are in bold.								

E

B+pre	.	4.58 (-13.49; 22.65)	.	.	.	.		
-1.83 (-30.00; 26.35)	L+B	-15.80 (-46.09; 14.49)	.	28.19 (2.54; 53.84)	.	.		
4.58 (-13.49; 22.65)	6.40 (-15.21; 28.02)	Pla	-31.60 (-57.63; -5.57)	-1.25 (-18.40; 15.89)	-0.75 (-12.43; 10.93)	-16.43 (-33.31; 0.44)		
-18.26 (-48.07; 11.55)	-16.43 (-48.52; 15.65)	-22.84 (-46.55; 0.87)	Pre	.	13.60 (-12.25; 39.45)	.		
10.44 (-13.53; 34.40)	12.26 (-8.39; 32.91)	5.86 (-9.88; 21.60)	**28.70 (0.24; 57.16)**	Ref	.	.		
3.83 (-17.69; 25.34)	5.66 (-18.91; 30.22)	-0.75 (-12.43; 10.93)	22.09 (-1.58; 45.76)	-6.61 (-26.21; 12.99)	Ref+L+B	.		
-11.86 (-36.58; 12.87)	-10.03 (-37.45; 17.39)	-16.43 (-33.31; 0.44)	6.40 (-22.70; 35.51)	-22.29 (-45.37; 0.78)	-15.69 (-36.21; 4.84)	Ref+L+Pre		
The upper triangle contains the pooled effect sizes of the direct comparisons available in our network. The lower triangle of the matrix contains the estimated effect sizes for each comparison. Significant results are in bold.								

F

B+pre	.	-22.11 (-50.11; 5.89)	.	.	.	.	.	
-22.21 (-58.82; 14.39)	L+B	9.95 (-16.46; 36.37)	.	-29.77 (-72.35; 12.81)	.	.	.	
-22.11 (-50.11; 5.89)	0.10 (-23.48; 23.68)	Pla	50.61 (-11.77; 112.99)	8.81 (-21.61; 39.22)	2.19 (-18.36; 22.74)	13.17 (-5.92; 32.26)	16.59 (-11.78; 44.96)	
-10.13 (-61.34; 41.08)	12.08 (-36.85; 61.02)	11.98 (-30.89; 54.86)	Pre	.	-0.57 (-41.75; 40.61)	.	.	
-26.37 (-64.58; 11.84)	-4.16 (-33.42; 25.11)	-4.26 (-30.26; 21.74)	-16.24 (-66.39; 33.90)	Ref	.	.	.	
-19.92 (-54.65; 14.81)	2.29 (-28.98; 33.57)	2.19 (-18.36; 22.74)	-9.79 (-49.53; 29.95)	6.45 (-26.69; 39.59)	Ref+L+B	.	.	
-8.94 (-42.83; 24.94)	13.27 (-17.07; 43.61)	13.17 (-5.92; 32.26)	1.19 (-45.75; 48.12)	17.43 (-14.83; 49.68)	10.97 (-17.07; 39.02)	Ref+L+B+Pre	.	
-5.52 (-45.38; 34.34)	16.69 (-20.20; 53.58)	16.59 (-11.78; 44.96)	4.61 (-46.81; 56.02)	20.85 (-17.64; 59.33)	14.40 (-20.63; 49.42)	3.42 (-30.77; 37.61)	Ref+L+Pre	
The upper triangle contains the pooled effect sizes of the direct comparisons available in our network. The lower triangle of the matrix contains the estimated effect sizes for each comparison. Significant results are in bold.								

G

B	.	.	-0.22 (-0.90; 0.46)	.	.	.	.	.
-0.54 (-1.56; 0.49)	B+pre	.	0.32 (-0.45; 1.08)	.	.	.	.	.
0.31 (-1.53; 2.15)	0.85 (-1.02; 2.72)	L+B	0.00 (-3.05; 3.05)	.	-0.51 (-2.20; 1.18)	.	.	.
-0.22 (-0.90; 0.46)	0.32 (-0.45; 1.08)	-0.53 (-2.24; 1.18)	Pla	0.24 (-0.11; 0.59)	0.26 (-0.92; 1.44)	0.37 (0.03; 0.71)	2.60 (2.29; 2.91)	0.50 (-1.08; 2.08)
0.01 (-0.75; 0.76)	0.54 (-0.29; 1.37)	-0.31 (-2.04; 1.43)	0.23 (-0.10; 0.55)	Pre	.	0.15 (-0.16; 0.46)	.	.
-0.04 (-1.35; 1.27)	0.50 (-0.85; 1.85)	-0.35 (-1.85; 1.15)	0.18 (-0.93; 1.30)	-0.04 (-1.21; 1.12)	Ref	.	.	.
0.15 (-0.61; 0.91)	0.68 (-0.15; 1.52)	-0.16 (-1.90; 1.58)	**0.37 (0.03; 0.71)**	0.14 (-0.16; 0.44)	0.18 (-0.98; 1.35)	Ref+L+B	.	.
**2.38 (1.63; 3.13)**	**2.92 (2.09; 3.74)**	**2.07 (0.33; 3.80)**	**2.60 (2.29; 2.91)**	**2.37 (1.92; 2.83)**	**2.42 (1.26; 3.58)**	**2.23 (1.77; 2.69)**	Ref+L+B+pre	.
0.28 (-1.44; 2.00)	0.82 (-0.94; 2.57)	-0.03 (-2.36; 2.29)	0.50 (-1.08; 2.08)	0.27 (-1.34; 1.89)	0.32 (-1.62; 2.25)	0.13 (-1.48; 1.75)	**-2.10 (-3.71; -0.49)**	Ref+L+Pre
The upper triangle contains the pooled effect sizes of the direct comparisons available in our network. The lower triangle of the matrix contains the estimated effect sizes for each comparison. Significant results are in bold.								

H

B+pre	-0.80 (-2.59; 0.99)	.	.	.				
-0.80 (-2.59; 0.99)	Pla	-1.51 (-2.62; -0.41)	-1.16 (-1.92; -0.40)	0.09 (-1.25; 1.43)				
**-2.31 (-4.42; -0.21)**	**-1.51 (-2.62; -0.41)**	Ref+L+B	.	.				
**-1.96 (-3.91; -0.01)**	**-1.16 (-1.92; -0.40)**	0.35 (-0.99; 1.69)	Ref+L+B+Pre	.				
-0.71 (-2.95; 1.53)	0.09 (-1.25; 1.43)	1.60 (-0.13; 3.34)	1.25 (-0.29; 2.79)	Ref+L+Pre				
The upper triangle contains the pooled effect sizes of the direct comparisons available in our network. The lower triangle of the matrix contains the estimated effect sizes for each comparison. Significant results are in bold.								

Supplementary Figure S5. Network alliance plot of different probiotics for (A) alanine aminotransferase (ALT), (B) aspartate transaminase (AST), (C) gamma glutamyl transferase (GGT), (D) fasting blood glucose (FBG), (E) total cholesterol (TC), (F) triglycerides (TG), (G) body Mass Index (BMI) and (H) fibroscan CAP score. The upper triangle in the matrix contains the pooled effect sizes of the direct comparisons in our network. The lower triangle in the matrix contains the estimated effect sizes for each comparison. Values in the matrix represent odds relative with 95% CI. If the odds ratio (OR)>0 or OR<0 and the confidence interval does not include 0, it indicates that one treatment is significantly more effective than the other. B: *Bifidobacterium*; L: *Lactobacillus*; Pla: *control group*; Pre: *prebiotics*; Ref: *Streptococcus thermophilus* and *Lactobacillus bulgaricus*.

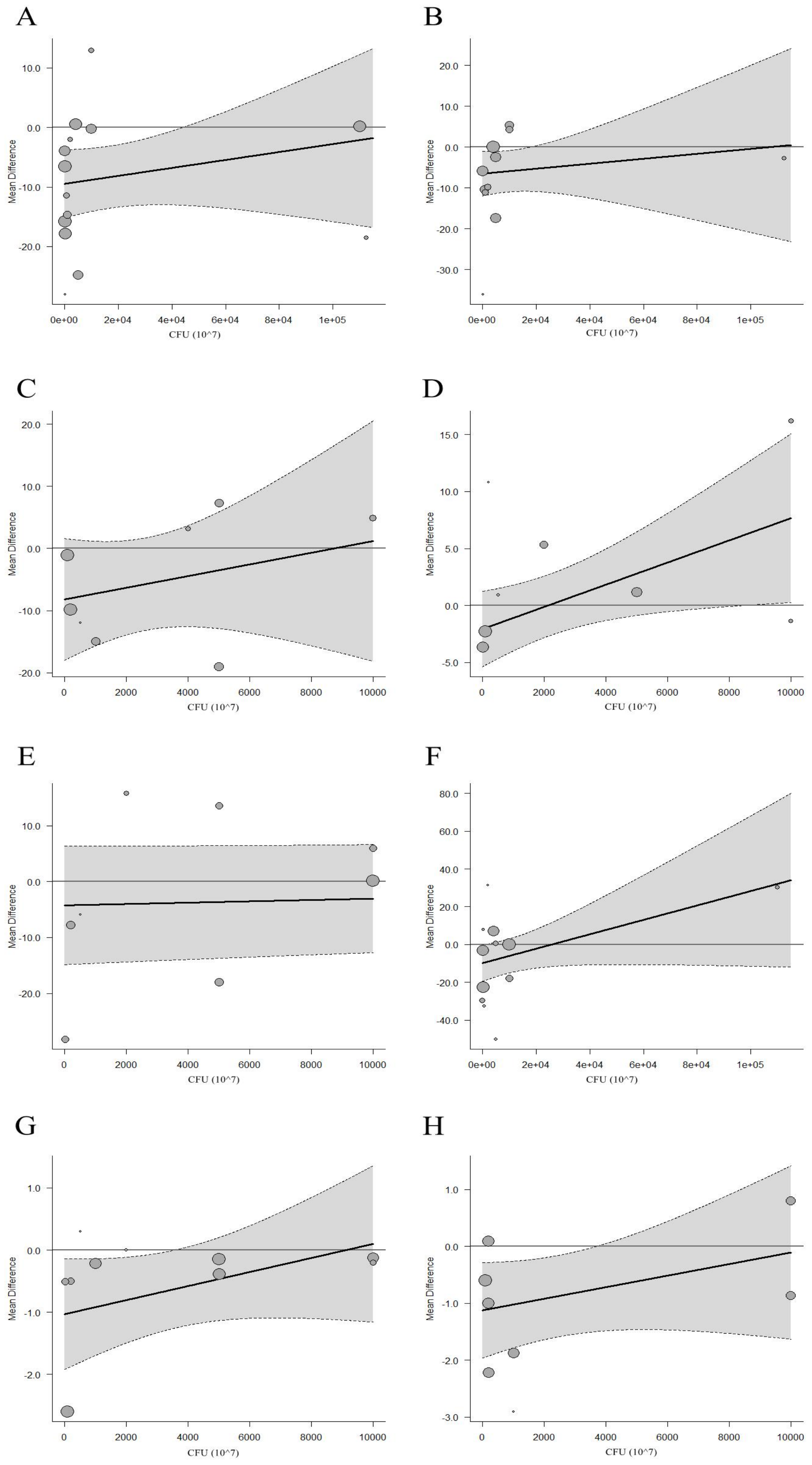

Supplementary Figure S6. Meta-regression analysis of CFU and mean difference of (A) alanine aminotransferase (ALT), (B) aspartate transaminase (AST), (C) gamma glutamyl transferase (GGT), (D) fasting blood glucose (FBG), (E) total cholesterol (TC), (F) triglycerides (TG), (G) body Mass Index (BMI) and (H) fibroscan CAP score. Circles mean the studies included in the analysis. The change in mean and 95% CI are represented by the solid and dotted lines, respectively. CFU: Colony Forming Units.

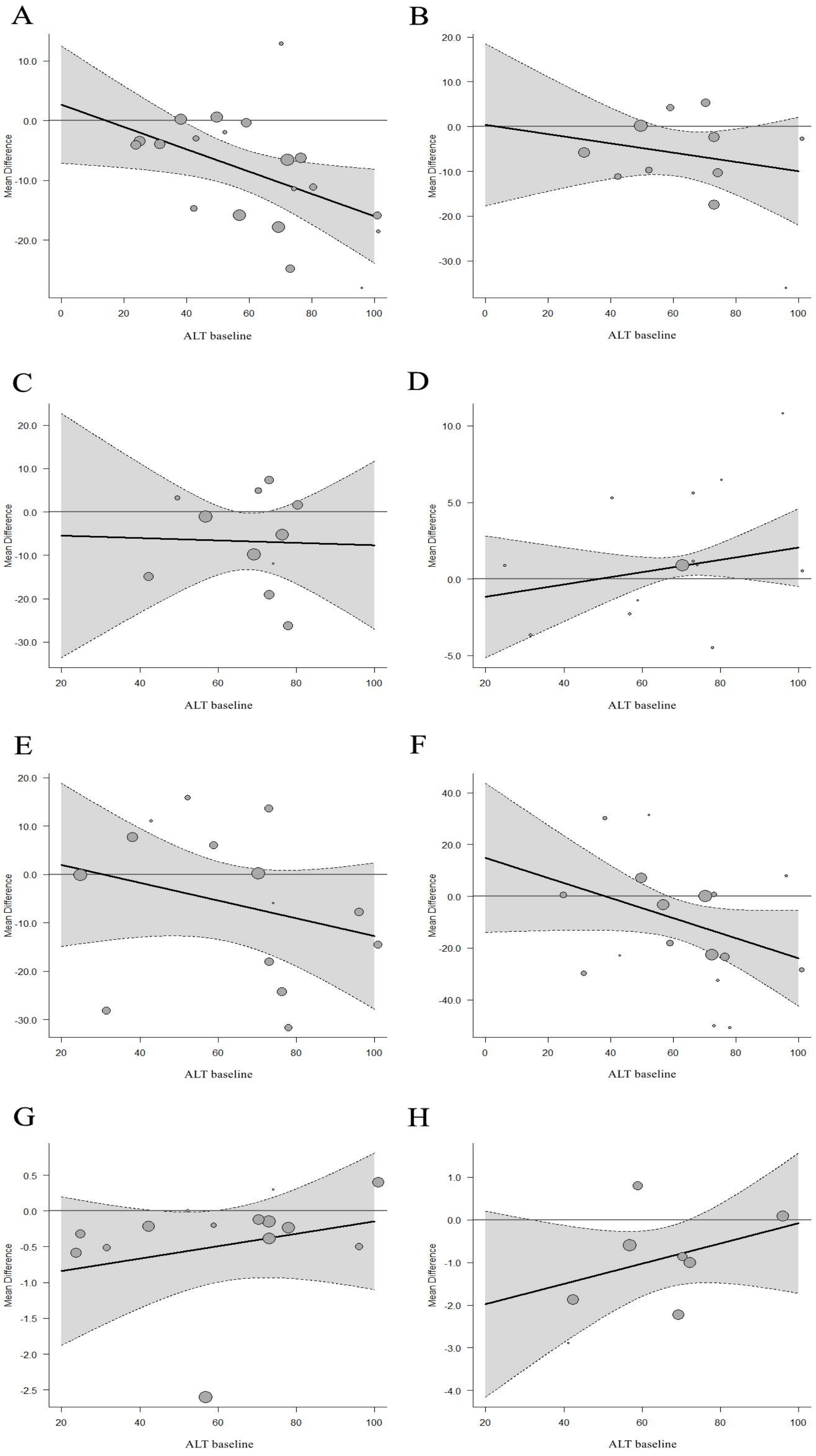

Supplementary Figure S7. Meta-regression analysis of the effect of ALT baseline on (A) alanine aminotransferase (ALT), (B) aspartate transaminase (AST), (C) gamma glutamyl transferase (GGT), (D) fasting blood glucose (FBG), (E) total cholesterol (TC), (F) triglycerides (TG), (G) body Mass Index (BMI) and (H) fibroscan CAP score. Circles mean the studies included in the analysis. The change in mean and 95% CI are represented by the solid and dotted lines, respectively.

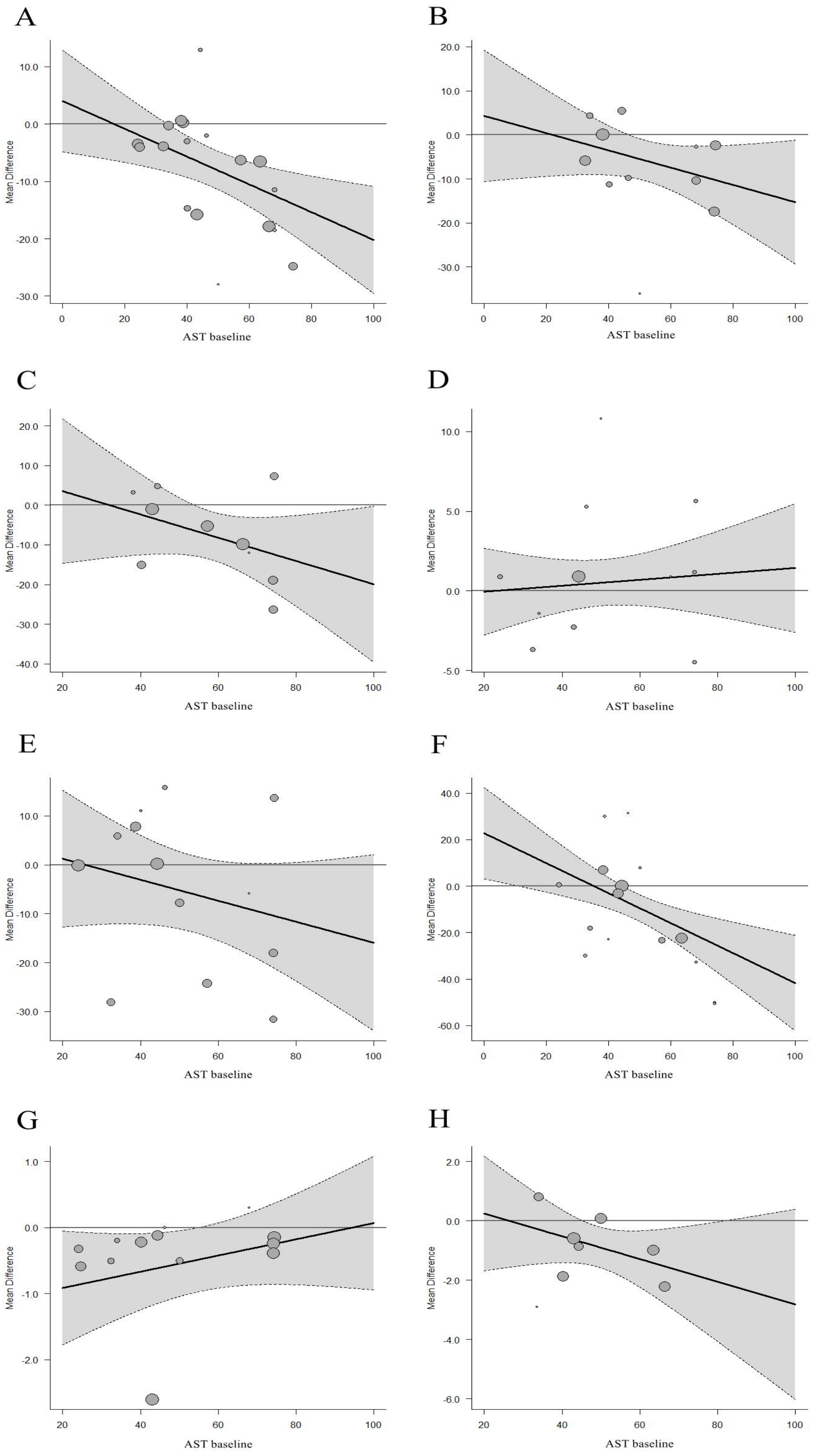

Supplementary Figure S8. Meta-regression analysis of the effect of AST baseline on (A) alanine aminotransferase (ALT), (B) aspartate transaminase (AST), (C) gamma glutamyl transferase (GGT), (D) fasting blood glucose (FBG), (E) total cholesterol (TC), (F) triglycerides (TG), (G) body Mass Index (BMI) and (H) fibroscan CAP score. Circles mean the studies included in the analysis. The change in mean and 95% CI are represented by the solid and dotted lines, respectively.

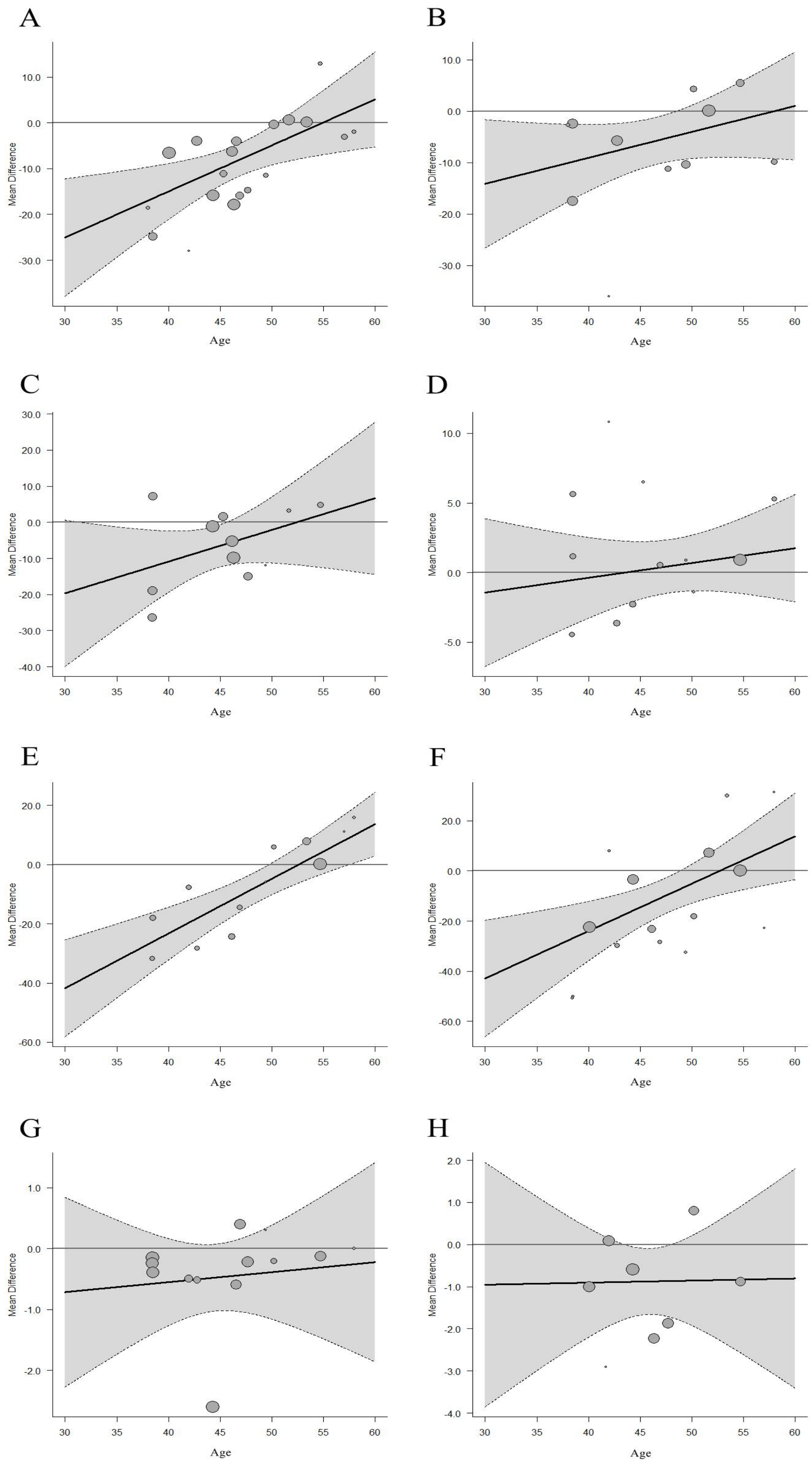

Supplementary Figure S9. Meta-regression analysis of the effect of Age on (A) alanine aminotransferase (ALT), (B) aspartate transaminase (AST), (C) gamma glutamyl transferase (GGT), (D) fasting blood glucose (FBG), (E) total cholesterol (TC), (F) triglycerides (TG), (G) body Mass Index (BMI) and (H) fibroscan CAP score. Circles mean the studies included in the analysis. The change in mean and 95% CI are represented by the solid and dotted lines, respectively.

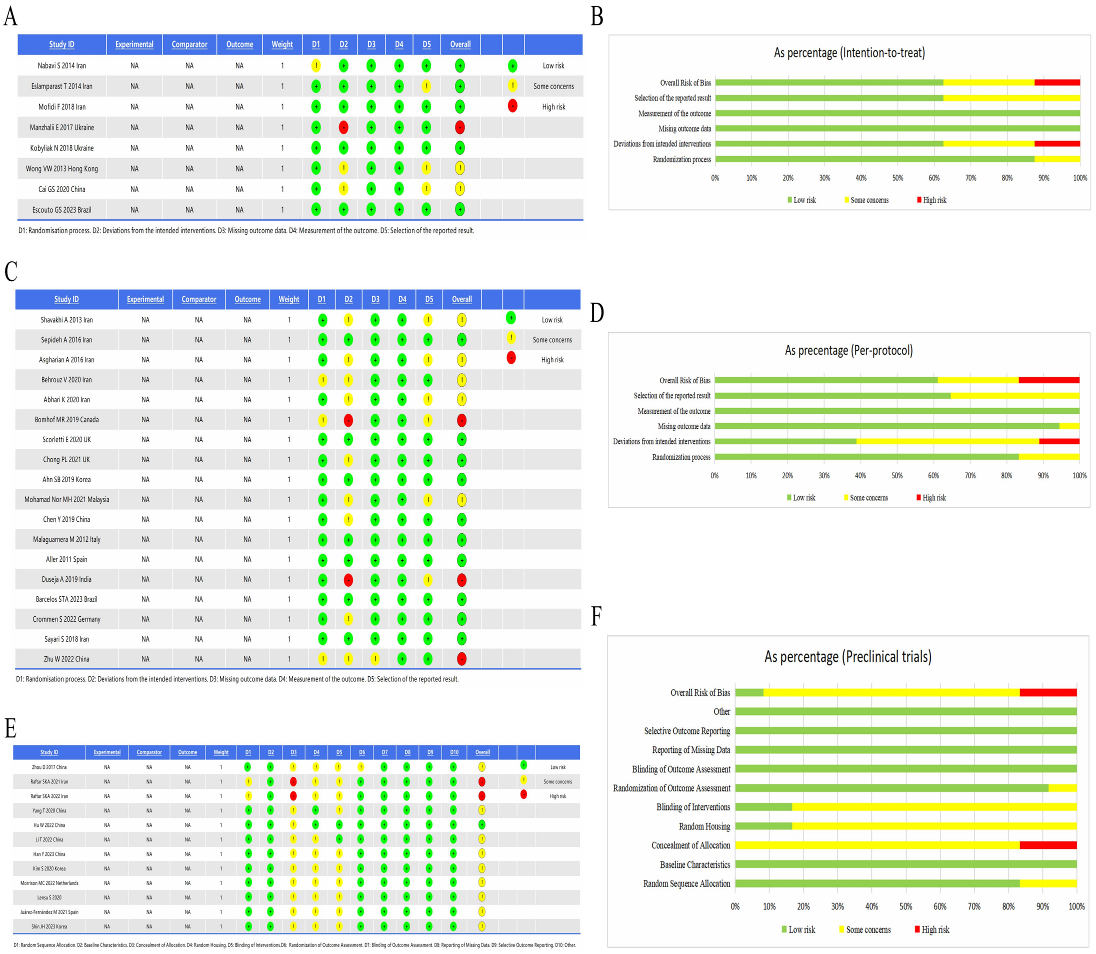

Supplementary Figure S10. Risk of bias analysis was performed on the 38 included research studies with the ROB 2 tool and SYRCLE ROB tool. The low risk of bias is presented in green, some concerns is presented in yellow, and the high risk of bias is presented in red. A. risk of bias summary tables for Intention-to-Treat studies. B. risk of bias as a percentage for Intention-to-Treat studies. C. Risk of bias summary tables for Per-Protocol studies. D. risk of bias as a percentage for Per-Protocol studies. E. Risk of bias summary tables for preclinical trials. F. risk of bias as a percentage for preclinical trials.
