# Supplementary material for: Roles of Traditional and Next-Generation Probiotics on Non-Alcoholic Fatty Liver Disease (NAFLD) and Non-Alcoholic Steatohepatitis (NASH): A Systematic Review and Network Meta-Analysis
Source: Antioxidants (Basel). 2024 Mar 7;13(3):329. doi: 10.3390/antiox13030329 (PMC10968178; doi:10.3390/antiox13030329)
Supplement: Supplementary file 1 [file antioxidants-13-00329-s001.zip › Supplementary table 09012024.pdf]

Supplementary Table S1 Baseline characteristics of the included clinical studies

Reference	Country	Study population	No. of participants who completed study	Study design	Probiotic/Synbiotic	Dose	Duration (W)	Post-intervention outcomes	Study objective
Shavakhi A 2013	Iran	Men and women (18-75 years)	63	Double-blind, randomized, placebo-controlled crossover study	*L. acidophilus* (10 million), *L. casei* (50 million), *L. rhamnosus* (7.5 million), *L. bulgaricus* (15 million) ,*B. breve* (5 million), *B. longum* (2.5 million), *S. thermophilus* (5 million), fructooligosaccharides 350mg. Control: protein similarities	2 tablets/day	24	AST↓, TC↓, TG→, BMI→, FBS→	Probiotic combination with Metformin improves liver aminotransferases better than metformin alone in patients with NASH.
Nabavi S 2014	Iran	Men and women (23-63 years)	72	Double-blind, randomized, placebo-controlled crossover study	Probiotic yogurts: *L. acidophilus* La5 (4.42 million), *B. lactis* Bb12 (3.85million). Control yogurts: *L. bulgaricus* (2.39million), *S. thermophilus* (2.08 million)	300g/day	8	ALT↓, AST↓, TC↓, TG↓, LDL-C↓,HDL-C→	Probiotic yogurt consumption improved hepatic enzymes, serum total cholesterol, and low-density lipoprotein cholesterol levels in studied subjects and might be useful in the management of NAFLD risk factors.
Eslamparast T 2014	Iran	Men and women (18-55 years)	52	Double-blind, randomized, placebo-controlled crossover study	Total (200 million): *L. casei*, *L. rhamnosus*, *S. thermophilus*, *B. breve*, *L. acidophilus*, *B. longum*, *L. bulgaricus*, fructooli-gosaccharide. Control: maltodexterin	2*109 CFU of each capsule/day	28	ALT↓, AST↓, GGT↓, CRP↓, TNF-α↓,NF-kB p65↓, Fibroscan CAP score↓	Synbiotic supplementation in addition to lifestyle modification is superior to lifestyle modification alone for the treatment of NAFLD, at least partially through attenuation of inflammatory markers in the body.
Sepideh A 2016	Iran	Men and women (18-75 years)	42	Double-blind, randomized, placebo-controlled crossover study	*L. casei* (3000 million), *L. acidophilus* (30000 million), *L. rhamnosus* (7000 million), *L. bulgaricus* (500 million), *B. breve* (20000 million), *B. longum* (1000 million), *S. thermophilus* (300 million). Control: maltodextrin, lactose, and magnesium stearate	1g of capsule/day	8	FBG↓, Insulin↓, HOMA-IR↓, IL-6↓, TNF-α↓, HbA1C↓	The effects of probiotic supplementation on the reduction of glycemic and inflammatory indices in patients with NAFLD.
Asgharian A 2016	Iran	Men and women (18-60 years)	74	Double-blind, randomized, placebo-controlled crossover study	*L. casei* (NS), *L. acidophilus* (NS), *L. rhamnosus* (NS), *L. bulgaricus* (NS), *B. breve* (NS), *B. longum* (NS), *S. thermophilus* (NS). Control: starch	500mg of each capsule/day	8	ALT→, AST→, CRP→	Symbiotic supplementation improved steatosis in NAFLD patients.
Mofidi F 2018	Iran	Men and women (≥18 years)	50	Double-blind, randomized, placebo-controlled crossover study	Total (200 million): *L. casei*, *L. rhamnosus*, *S. thermophilus*, *B. breve*, *L. acidophilus*, *B. longum*, *L. bulgaricus*, fructo-oligosaccharide 125mg, magnesium stearate. Control: maltodexterin	2 tablets/day	28	TG↓, LDL-C↓, TG↓, CRP↓, HDL-C→, TNF-α→, NF-κB p65↓, FBS→, Insulin→, HOMA-IR→, QUICKI→, Fibroscan CAP score↓	Synbiotic supplementation improves the main features of NAFLD in patients with normal and low BMI, at least partially through the reduction in inflammatory indices.
Behrouz V 2020	Iran	Men and women (20-60 years)	89	Parallel, double-blind, randomized, placebo-controlled crossover study	Total (5000 million): *L. casei*, *L. rhamnosus*, *L. acidophilus*, *B. longum*, *B. breve*, fructo-oligosaccharide. Control: Starch, maltodextrin	2 capsules/day	12	ALT↓, AST↓, TC↓, TG↓, GGT↓, ALP↓, LDL-C↓, FBS↓	Probiotics and prebiotics may be beneficial in improving liver enzymes and lipid profiles in patients with NAFLD.
Abhari K 2020	Iran	Men and women (18-75 years)	46	Double-blind, randomized, placebo-controlled crossover study	*Bacillus coagulans (GBI-30)* (1000 million), Control: maltodexterin	1 capsule/day	12	ALT↓, GGT↓, TNF-α↓, NF-κB↓, Insulin↓, Fibroscan CAP score ↓	*Bacillus coagulans* is beneficial for the treatment of NAFLD and its related inflammation without any significant effects on related cardiovascular risk factors.
Bomhof MR 2019	Canada	Men and women (≥18 years)	14	Double-blind, randomized, placebo-controlled crossover study	fructo-oligosaccharide. Control: Starch, maltodextrin	8g 12w/day<br>16g 24w/day	36	TC↓, TG↓, NAS↓, liver fibrosis score→, ALT→, GGT→, ALP →, IL-6→, TNF-α→, Insulin→	Independent of other lifestyle changes, prebiotic supplementation reduced histologically confirmed steatosis<br>in patients with NASH.
Scorletti E 2020	UK	Men and women (≥18 years)	104	Double-blind, randomized, placebo-controlled crossover study	*B. animalis subsp. lactis* BB-12 (10000 million), fructo-oligosaccharide 4g. Control: maltodextrin	1 capsule/day	48	TC↓, TG↓, NAS↓, Fibroscan CAP score→	Synbiotic treatment was effective in changing gut microbiota but this pro- and prebiotic combination was ineffective in improving liver fat content or liver fibrosis markers in NAFLD.
Chong PL 2021	UK	Men and women (25-70 years)	35	Double-blind, randomized, placebo-controlled crossover study	Total (4500 million): *S. thermophilus*, *B. breve*, *B. longum*, *B. infantis*, *L. acidophilus*, *L. plantarum*, *L. paracasei*, *L. bulgaricus*. Control: maltodextrin	2 sachets/day	10	ALT→, AST→, sVCAM-1→, cGMP→, glutathione ratio→, LHP→, CRP→, HOMA-IR→, ASQ→	Probiotics did not significantly improve markers of cardiovascular risk and liver injury in patients with NAFLD. However, the study supports an association between endothelial dysfunction and inflammation in patients with NAFLD and suggests that NAFLD is linked with insulin resistance.
Manzhalii E 2017	Ukraine	Men and women (≥18 years)	75	Randomized, controlled, non-blinded, prospective clinical trial	Total (100 million): *L. casei*, *L. rhamnosus*, *L. bulgaris*, *B. longum*, *S. thermophilus*, fructooligosaccharides. Control: low-fat/low-calorie diet	1 capsule/day	12	ALT↓, AST↓, GGT→, TC↓, BMI↓, Fibroscan CAP score↓	Short-term treatment with the probiotic cocktail caused<br>significant improvement of liver inflammation status without adverse events.
Kobyliak N 2018	Ukraine	Men and women (≥18 years)	58	Parallel, double-blind, randomized, placebo-controlled crossover study	*L.+ Lactococcus* (60000 million), *B.* (10000 million), *Propionibacterium* (30000 million), *Acetobacter genera* (1 million). Control: sachets	1 sachet/day	8	AST↓, TC↓, TG↓, LDL-C↓, VLDL↓, TNF-α↓, IL-6↓, FLIFL↓, VAI↓, LS→	The probiotic "Symbiter" reduces liver fat, aminotransferase activity, and TNF-α and IL-6 levels in NAFLD patients.
Ahn SB 2019	Korea	Men and women (19-75 years)	65	Randomized, controlled, non-blinded, prospective clinical trial	Total (1000 million): *L. acidophilus*, *L. rhamnosus*, *L. paracasei*, *Pediococcus pentosaceus*, *B. lactis*, *B. breve*. Control: dextran, maltodextrin, lemon favor, and Mg stearate	1 capsule/day	12	Body weight↓, BMI↓, total body fat mass↓, percentage of total body fat↓, visceral fat grade↓, TC↓, TG↓, TNF-α↓, Fibroscan CAP score↓	Treatment with probiotics for 12 weeks resulted in a significant reduction in IHF and body weight in obese NAFLD patients.
Mohamad Nor MH 2021	Malaysia	Men and women (≥18 years)	39	Double-blind, randomized, placebo-controlled crossover study	Total (30000 million): *L. acidophilus* BCMC 12,130 (107 mg), *L. casei subsp.* BCMC 12,313 (107 mg), *L. lactis* BCMC 12,451 (107 mg), *B. bifidum* BCMC 02290 (107 mg), *B. infantis* BCMC 02129 (107 mg), *B. longum* BCMC 02120 (107 mg). Control: sachets	1 sachet/day	24	ALT→, AST→, GGT→, TG→, TC→, FBG→, CD4+ T lymphocytes→, CD8+ T lymphocytes→, CAP→, Fibroscan CAP score→	Probiotics seemed to be able to stabilize the mucosal immune function and protect NAFLD patients against increased intestinal permeability.
Wong VW 2013	Hong Kong	Men and women (≥18 years)	20	Open-label, randomized, placebo-controlled crossover study	Total (200 million): *L. plantarum* ATCC 14917, *L. deslbrueckii ssp. bulgaricus* ATCC 11842, *L. acidophilus* (ATCC 4366, *L. rhamnosus* ATCC 7469, *B. bifidum* ATCC 29521, fructo-oligosaccharide (3g).Control： General treatment modalities	2 sachets/day	24	Fibroscan CAP score→	Probiotic treatment may reduce liver fat and AST levels in NASH patients.
Chen Y 2019	China	Women（36-66 years）	92	Double-blind, randomized, placebo-controlled crossover study	Yogurt: *L. delbrueckii ssp. bulgaricus* and *S. thermophiles*. Control: milk	220g/day	24	ALT↓, TC↓, TG↓, LPS↓,HFF↓, IHL↓, HOMA-IR↓, Insulin↓, FBG ↓, FGF21↓, GSH-Px↑, SOD↑	Yogurt was better than milk at ameliorating HOMA-IR and liver fat in obese Chinese women with NAFLD and Met S, possibly by improving lipid metabolism, reducing inflammation, oxidative stress, and LPS, and changing the gut microbiota composition.
Cai GS 2020	China	Men and women (18-59 years)	140	Double-blind, randomized, placebo-controlled crossover study	Live Combined *B.*, *L.* and Enterococcus Powder. Control: General treatment modalities	2g/day	12	ALT↓, AST↓, TC↓, TG↓, GGT↓, HOMA-IR↓, LDL-C↓, NAS↓, HDL-C↑, TBIL→	Probiotics can improve some liver functions, glucose and lipids metabolism, hepatic fatty deposition in patients with NAFLD, which will enhance the therapeutic effects of NAFLD.
Malaguarnera M 2012	Italy	Men and women (≥18 years)	63	Double-blind, randomized, placebo-controlled crossover study	*B. longum* (NS), fructo-oligosaccharide. Control: placebo	1 sachet/day	24	AST↓, LDL-C↓, CRP↓, TNF-α↓, HOMA-IR↓, NAS↓	*B. longum* with Fos and lifestyle modification, when compared to lifestyle modification alone, significantly reduces TNF-a, CRP, serum AST levels, HOMA-IR, serum endotoxin, steatosis, and the NASH activity index.
Aller R 2011	Spain	Men and women (≥18 years)	28	Double-blind, randomized, placebo-controlled crossover study	Total (500 million): *L. bulgaricus*, *S. thermophiles*. Control: placebo (120mg)	1 tablet/day	12	ALT↓, AST↓, GGT↓, LDL-C→, HDL-C→, Insulin levels→, HOMA-IR→, IL-6→, TNF-α→	500 million of *L. bulgaricus* and *S. thermophilus*, with a randomized clinical design, improved liver aminotransferase levels in patients with NAFLD.
Duseja A 2019	India	Men and women (≥18 years)	30	Double-blind, randomized, controlled crossover study	Total (112500 million): *L. paracasei* DSM 24733, *L. plantarum* DSM 24730, *L. acidophilus* DSM 24735, *L. delbrueckii subsp. bulgaricus* DSM 24734, *B. longum* DSM 24736, *B. infantis* DSM 24737, *B. breve* DSM 24732, *S. thermophilus* DSM 24731. Control: microcrystalline cellulose	6 capsules/day	48	ALT↓, TNF-α↓, IL-1β↓,IL-6↓, HOMA-IR→, SIBO→, NAS↓	Patients with NAFLD managed with lifestyle modifications and multistrain probiotic showed significant improvement in liver histology, ALT and cytokines.
Escouto GS 2023	Brazil	Men and women (≥18 years)	48	Parallel, double-blind, randomized, controlled crossover study	*L. acidophilus* ATCC SD5221(1000 million), *B. lactis* HN019 (1000million). Control: maltodextrin	1 capsule/day	24	ALT↓, AST→, TC→, TG→, LDL-C→, HDL-C→, HbA1cs→, HOMA-IR→, CRP→, FLI→, APRI↓	Patients with NASH who received probiotics supplementation for 6 months presented improvement in the APRI score after treatment, but supplementation with probiotics alone is not sufficient to improve enzymatic liver markers, inflammatory parameters, and gut microbiota in patients with NASH.
Barcelos STA 2023	Brazil	Men and women (≥18 years)	44	Triple-blind, randomized, controlled crossover study	*L. acidophilus* NCFM (1000million), *L. rhamnosus* HN001 (1000million), *L. paracasei* LPC-37 (1000million), *B. lactis* HN019 (1000million). Control: polydextrose/maltodextrin	2 sachet/day	24	Castelli's Risk Index (CRI)↓, Atherogenic Coefficient (AC)↓, PAI-1↓, miR-122↓, BMI→, CRP→, CK→	A 24-week probiotic mix administration was not superior to placebo in reducing CVR markers in patients with NASH.
Crommen S 2022	Germany	Men and women (20-65 years)	48	Parallel, double-blind, randomized, controlled crossover study	Total (1500 million): *L. acidophilus*, *B. breve*, *B. longum*, *L. delbrueckii susp. bulgaricus*, *L. helveticus*, *L. plantarum*, *L. rhamnosus*, *L. casei*, *L. lactis susp. lactis*, *S. thermophiles.* Control: corn dextrin and rice starch	4g/day	12	ALT↓, AST→, TC→, TG↓, LDL-C→, HDL-C↑, HbA1cs→, HOMA-IR→, CRP→, FLI→, VAI↓, NAS↓	Supplementation with a specifically tailored probiotic and micronutrient mixture improved NAFLD-related markers more than a basic micronutrient mixture in obese patients following mini gastric bypass (MGB) surgery.
Sayari S 2018	Iran	Men and women (18-60 years)	138	Parallel, double-blind, randomized, controlled crossover study	*L. casei*, *L. rhamnosus*, *L. acidophilus*, *L. bulgaricus*, *B. breve*, *B. longum*, *S. thermophilus*, fructo-oligosaccharide, magnesium stearate. Control: maltodexterin	500mg of each capsule/day	16	ALT↓, TG↓, TC→, HDL-C→, LDL-C↓, FBS↓	Sitagliptin-synbiotic produced greater improvement in FBS, AST, Cholesterol, and LDL compared to sitagliptin alone in patients with NAFLD.

Zhu W 2022	China	Men and women (≥18 years)	96	Double-blind, randomized	*Rosuvastatin+Clostridium butyricum* (1200mg)<br>Control: Rosuvastatin	1200g/day	24	ALT↓, AST↓, TBIL↓, DBIL↓, TC↓, TG↓, FFA↓, PIIIP↓, C-IV↓, HA ↓, LN↓, TNF-α↓, CRP↓, IL-6↓	*Clostridium butyricum* combined with *Rosuvastatin* can more effectively improve gut microbiota imbalance, reduce blood lipid levels, alleviate liver fibrosis, and lessen liver function damage in patients with NAFLD.

AC: Atherogenic Coefficient; ALP: Alkaline Phosphatase; ALT: Alanine Aminotransferase; APRI: AST to Platelet Ratio Index; ASQ: Autism Spectrum Quotient; AST: Aspartate Aminotransferase; *B.: Bifidobacterium*; BMI: Body Mass Index; CAP: Controlled Attenuation Parameter; C-IV: Collagen Type IV; CK: Creatine Kinase; CRP: C-Reactive Protein; CRI: Castelli's Risk Index; cGMP: Cyclic Guanosine Monophosphate; CVR: cardiovascular risk; DBIL: Direct Bilirubin; FBG: Fasting Blood Glucose; FGF21: Fibroblast Growth Factor 21; FLIFL: Fatty Liver Infiltration and Fibrosis Score; Fibroscan CAP score: FibroScan Controlled Attenuation Parameter score; GGT: Gamma-Glutamyl Transferase; GSH-Px: Glutathione Peroxidase; HbA1C: Hemoglobin A1C; HDL-C: High-Density Lipoprotein Cholesterol; HFF: Hepatic Fat Fraction; HOMA-IR: Homeostatic Model Assessment of Insulin Resistance; IHL: Intrahepatic Lipid; IL-1β: Interleukin 1 Beta; IL-6: Interleukin 6; *L.: Lactobacillus*; LDL-C: Low-Density Lipoprotein Cholesterol; LHP: Lipid Hydroperoxides; LN: Laminin;LPS: Lipopolysaccharides; LS: Liver Stiffness; MGB: Mini Gastric Bypass; Met S: Metabolic Syndrome; NAFLD: Non-Alcoholic Fatty Liver Disease; NAS: NAFLD Activity Score; NASH: Non-Alcoholic Steatohepatitis; NF-kB p65: Nuclear Factor Kappa-light-chain-enhancer of activated B cells p65; PAI-1: Plasminogen Activator Inhibitor-1; PIIIP: Procollagen III N-terminal Peptide; QUICKI: Quantitative Insulin Sensitivity Check Index; *S.: Streptococcus*; SIBO: Small Intestinal Bacterial Overgrowth; SOD: Superoxide Dismutase; TBIL: Total Bilirubin; TC: Total Cholesterol; TG: Triglycerides; TNF-α: Tumor Necrosis Factor Alpha;VLDL: Very Low-Density Lipoprotein; VAI: Visceral Adiposity Index; ↑: increased; ↓: decreased; ⟶: no change

Supplementary Table S2 Baseline characteristics of the included preclinical studies

Reference	Country	Animal model	No. of animals	Sex/age (wk)	Weight change	No. of groups	No. of animals per group	CRC induction	Control group	Probiotic/synbiotic	Method of administration	Daily dose	Duration of intervention (wk)	Post-intervention outcomes	Study objective
Zhou D 2017	China	C57BL/6 mice	30	male	decline	3	10	HFD, for 16 wk	HFD+PBS	*Clostridium butyricum* B1	Oral gavage	10^9 CFU/g of body weight	8	ALT↓, AST↓, FBG↓, HOMA-IR↓, TNF-α↓, MCP-1↓, IL-1β↓, IL-2↓, IL-6↓, IL-10↓, IFN-γ↓, IL-17↓, Foxp3↑, IL-4↑, IL-22↑	Clostridium butyricum B1 could attenuate HFD-induced steatohepatitis in mice partially through butyrate-induced enterohepatic immunoregulation.
Yang T 2020	China	C57BLKS/J (db/db) mice	27	male	NS	4	8/6/7/6	normal diet	normal diet	*Clostridium butyricum*	Oral gavage	5*10^7 CFU/Kg	6	ALT↓, AST↓, TG↓, ALP↓, LDLC↓, T-CHO↓, IL-1β ↓, IL-6↓, TNF-α↓, ZO-1(+)↑, occludin(+)↑, TGR5↑, GLP-1↑	Clostridium butyricum exerts a hepatoprotective effect by promoting GLP-1 secretion through the increased expression of TGR5 protein mediated by intestinal tight junctions (TJs). It demonstrates potential therapeutic efficacy in reducing lipid accumulation and inflammation in NAFLD mouse models.
Raftar SKA 2021	Iran	C57BL/6 mice	25	male	NS	5	5	HFD+CCL4, for 4 wk	HFD+PBS	*A. muciniphila* MucT (ATCC BAA-835)	Oral gavage	10^9 CFU/200μl	4	ALT↓, AST↓, TC↓, TG↓, LDL↓, VLDL↓, TNF-a↓, IL-6↓, a-SMA↓, PDGF↓, TGF-b↓, TLR-2↓, TLR-4 ↓, TNF-a↓, L-10↑, HDL↑, SCFAs↑,	Oral administration of live and pasteurized A. muciniphila and its EVs could normalize the fecal targeted bacteria composition, improve the intestinal permeability, modulate inflammatory responses, and subsequently prevent liver injury in HFD/CCl4-administered mice.
Juárez-Fernández M 2021	Spain	Wistar rats	56	male	NS	8	7	HFD, for 6 wk	HFD+PBS	*A. muciniphila* (CIP-107961T)	Oral gavage	2*10^8 CFU/200μl	3	Primary bile acids↑, SCFAs↑, Cyp7a1 unchanged, Cyp8b1↑, Ntcp↑, Bsep↑, Mrp2↑, Fatp5↑, Baat↑, Lxrα↑, Fxr↑, Shp unchanged	A. muciniphila demonstrates potential positive effects in alleviating obesity features induced by a high-fat diet, modulating gut microbiota composition, increasing plasma bile acid levels, and influencing factors related to the gut microbiota, bile acids, and liver gene expression.
Raftar SKA 2022	Iran	C57BL/6J mice	25	male	NS	5	5	HFD+CCL4, for 4 wk	HFD+PBS	*A. muciniphila* MucT (ATCC BAA-835)	Oral gavage	10^9 CFU/200μl	4	IL-6↓, TNF-α↓, TLR-5↓, TLR-9↓, IL-1β↓, PPAR-α↑, PPAR-γ↑, IL-10↑	The present results showed that oral administration of A. muciniphila and its derivatives for four weeks could enhance the intestinal integrity and anti-inflammatory responses of the colon, adipose, and liver tissues and subsequently prevent liver injury in HFD/CCL4 mice.
Hu W 2022	China	C57BL/6J mice	30	male	decline	3	10	HFD	HFD+PBS	*F. prausnitzii* (A2-165, JZ10, JZ27, LA8, LB8, QL13, QL33, SM10, ZF21, HW29, PL45, and LC49)	Oral gavage	10^9 CFU of body	12	ALT↓, AST↓, FFAs↓, TC↓, TG↓, LDL-C↓, HDL-C ↑, fat tissue index↓, GSH-PX↑, SOD↑, MDA↓, adipocyte size↓, TNF-α↓, IL-6↓, FBG↓, fasting insulin↓, HOMA-IR↓, acetate↑, propionate↑, butyrate↑, iso-butyrate unchanged	F. prausnitzii LB8 and LC49 significantly ameliorated the symptoms associated with a mouse model of NAFLD, restored the gut microbial dysbiosis, and modulated the gut microbial functional pathways and SCFAs production.
Shin JH 2023	Korea	C57BL/6 mice	96	female	invariably	8	12	HFHF	HFHF	*F. prausnitzii* (A2-165, EB-FPDK3, EB-FPDK9, EB-FPDK11, EB-FPYYK1)	Oral (in diet)	10^8 CFU	9	TC↓, TG↓, TNF-α↓, TLR4↓, MCP-1↓, IL-6↓, CD36↓, FATP5↓, PPAR-γ↓, SREBP-1c↓, FAS↓, LPL↓, Col1a1↓, TIMP-1↓, ZO-1(+)↑, occludin(+) ↑	F. prausnitzii in the treatment of NASH is characterized by its anti-inflammatory effects, improvement of glucose homeostasis, restoration of gut barrier function, and reduction of liver damage through the activation of the Nrf2 pathway with butyric acid
Li T 2022	China	C57BL/6J mice	24	male	invariably	4	6	HFD+STZ， for 10 wk	HFD+PBS	*A. muciniphila* (AM06， AM02)	Oral gavage	10^9 CFU of body	10	Number and maximum diameter of liver tumor nodules↓, AST↓, IL-6↓	Administration of breast milk-isolated A. muciniphila (AM06) but not feces-isolated A. muciniphila (AM02) could improve NASH severity. Interestingly, breast milk-isolated A. muciniphila treatment suppressed the progression of NASH to HCC, accompanied with an increased hepatic CXCR6+ natural killer T (NKT) cell and decreased macrophage infiltration.
Han Y 2023	China	C57BL/6J mice	18	male	decline	3	6	HFD, for 20 wk	HFD+PBS	*A. muciniphila* MucT (ATCC BAA-835)	Oral gavage	10^9 CFU/200μl	20	ALT↓, AST↓, FBG↓, TC↓, hepatic γδT cells↓, hepatic γδT17 cells↓, hepatic proinflammatory M1 macrophages↓, hepatic TLR2↓, propionic↑, 2-methylbutyric↑, isovaleric↑, valeric↑.	A. muciniphila supplementation prevented hepatic inflammation in high-fat diet-induced NASH mice, characterized by reduced hepatic proinflammatory macrophages (M1) and γδT and γδ T17 cells. Furthermore, A. muciniphila inhibited intestinal barrier disruption and accordingly downregulated hepatic Toll-like receptor 2 (TLR2) expression in NASH mice. A.muciniphila prevented NASH by modulating TLR2-activated γδT17 cells and further macrophage polarization.
Kim S 2020	Korea	C57BL/6N mice	20	male	invariably	4	5	HFD, for 5 wk	HFD+PBS	*A. muciniphila* MucT (ATCC BAA-835)	Oral gavage	10^9 CFU/ml	10	ALT↓, AST↓, TG↓, SREBP↓, IL-6↓	Oral administration of A. muciniphila significantly (P < 0.05) lowered serum triglyceride (TG) and alanine aminotransferase (ALT) levels in obese mice. Compared to the non-A. muciniphila-treated group, the expression of SREBP (regulator of TG synthesis in liver tissue) was decreased in the A. muciniphila-treated group. The expression of IL-6 in the liver of obese mice was decreased following the administration of A. muciniphila. Furthermore, alterations in the ratio of Firmicutes to Bacteroidetes and the decrease in bacterial diversity caused by the HFD were restored upon the administration of A. muciniphila.
Morrison MC 2022	Netherlands	Ldlr.Leiden mice	36	male	invariably	3	15/15/6	HFD, for 28 wk	HFD	Heat-inactivated *A. muciniphila*	Oral (in diet)	2*10^8 CFU of body	28	Intestinal permeability↓, butyrate↓, valeric↓	Heat-inactivated A. muciniphila can improve obesity-induced gut permeability and associated basal lamina turnover markers (PRO-C4) and has minor effects on tissues in close proximity to the gut, i.e., mesenteric white adipose tissue hypertrophy.
Lensu S 2020	Finland	Wistar rats	40	male	NS	4	10	HFD	HFD	*F. prausnitzii* (ATCC 27766 and DSM A2-165) Xylo-oligosaccharides	Oral (in diet)	0.05 g/kg	12	TC↓, β-HAD↑,	XOS increased F. prausnitzii growth, having only a minor impact on the GM composition. When supplemented with HFD, XOS ameliorated hepatic steatosis. The underlying mechanisms involved enhanced hepatic β-oxidation and mitochondrial respiration.

A. muciniphila: Akkermansia muciniphila; ALP: Alkaline Phosphatase; ALT: Alanine Aminotransferase; AST: Aspartate Aminotransferase; Baat: Bile Acid-CoA: Amino Acid N-Acyltransferase; Bsep: Bile Salt Export Pump; CCl4: Carbon Tetrachloride; CD36;Col1a1: Collagen Type I Alpha 1 Chain; CFU: colony-forming unit; Cyp7a1: Cytochrome P450 7A1; Cyp8b1: Cytochrome P450 8B1; EVs: Extracellular Vesicles; *F. prausnitzii*: Faecalibacterium prausnitzii; FAS: Fatty Acid Synthase; Fatp5: Fatty Acid Transport Protein 5; FBG: Fasting Blood Glucose; FFAs: Free Fatty Acids; Foxp3: Forkhead Box P3; Fxr: Farnesoid X Receptor; GLP-1: Glucagon-Like Peptide-1; GSH-PX: Glutathione Peroxidase; HDL: High-Density Lipoprotein; HDL-C: High-Density Lipoprotein Cholesterol; HFD: High-Fat Diet; HOMA-IR: Homeostatic Model Assessment of Insulin Resistance; IFN-γ: Interferon Gamma; IL-1β: Interleukin 1 Beta; IL-2: Interleukin 2; IL-4: Interleukin 4; IL-6: Interleukin 6; IL-10: Interleukin 10; IL-17: Interleukin 17; IL-22: Interleukin 22; LDL: Low-Density Lipoprotein; LDL-C: Low-Density Lipoprotein Cholesterol; LPL: Lipoprotein Lipase; Lxrα: Liver X Receptor Alpha; MCP-1: Monocyte Chemoattractant Protein-1; MDA: Malondialdehyde; Mrp2: Multidrug Resistance Protein 2; Ntcp: Sodium Taurocholate Co-transporting Polypeptide; PDGF: Platelet-Derived Growth Factor; PPAR-α: Peroxisome Proliferator-Activated Receptor Alpha; PPAR-γ: Peroxisome Proliferator-Activated Receptor Gamma; SOD: Superoxide Dismutase; SREBP: Sterol Regulatory Element-Binding Protein; SREBP-1c: Sterol Regulatory Element-Binding Protein 1c; STZ: Streptozotocin; TC: Total Cholesterol; TG: Triglycerides; TGF-b: Transforming Growth Factor Beta; TIMP-1: Tissue Inhibitor of Metalloproteinases 1; TLR-2: Toll-Like Receptor 2; TLR-4: Toll-Like Receptor 4; TLR-5: Toll-Like Receptor 5; TLR-9: Toll-Like Receptor 9; TNF-α: Tumor Necrosis Factor Alpha; T-CHO: Total Cholesterol; TLR2: Toll-Like Receptor 2; VLDL: Very Low-Density Lipoprotein; XOS: Xylo-oligosaccharide; ZO-1(+): Zonula Occludens-1; ↑: increased; ↓: decreased; ⟶: no change

Supplementary Table S3 Subgroup analysis

Nation	Marker	MD	95% CI	P-value	I^2	P-subgroup
Iran	ALT	-11.399	-20.147, -2.651	0.020	95%	< 0.0001
Iran	AST	-10.339	-18.377, -2.302	0.023	64.6%	0.009
Iran	TC	-25.560	-43.413, -7.702	0.025	0%	< 0.0001
Iran	TG	-22.462	-26.423, -18.502	0.015	4.1%	< 0.0001
Iran	BMI	-0.254	-0.442, -0.066	0.013	0%	< 0.0001
Iran	Fibroscan CAP score	-1.638	-3.242, -0.033	0.047	66.1%	< 0.0001

ALT: Alanine Aminotransferase; AST: Aspartate Aminotransferase; BMI: Body Mass Index; CFU: colony-forming unit; TC: Total Cholesterol; TG: Triglycerides; Fibroscan CAP score: FibroScan Controlled Attenuation Parameter score

Supplementary Table S4 Meta-regression output

Model	ALT						AST						GGT						FBG					
No covariates	N	Coeff. (95% CI)	τ^2	Adj. R ^{2}%	I^2	p Value	N	Coeff. (95% CI)	τ^2	Adj. R ^{2}%	I^2	p Value	N	Coeff. (95% CI)	τ^2	Adj. R ^{2}%	I^2	p Value	N	Coeff. (95% CI)	τ^2	Adj. R ^{2}%	I^2	p Value
Univariate																								
CFU	14	0.0001 (-0.0001, 0.0002)	7.7720	5.03%	97.12%	0.3607	11	0.0001 (-0.0002, 0.0003)	6.5135	0	72.45%	0.6286	8	0.0009 (-0.0015, 0.0034)	8.0889	0	95.93%	0.4473	8	0.0010 (0.0001, 0.0019)	0	100%	0.00%	0.0304
ALT baseline	20	-0.1868 (-0.3500, -0.0235)	5.6976	25.54%	88.96%	0.0249	11	-0.1034 (-0.3858, 0.1790)	6.6438	0	70.37%	0.4730	11	-0.0276 (-0.5974, 0.5422)	8.8290	0	92.18%	0.9244	12	0.0371 (-0.0426, 0.1167)	0	100%	0	0.3233
AST baseline	19	-0.2422 (-0.4177, -0.0727)	5.3851	36.55%	86.91%	0.0051	11	-0.1958 (-0.4703, 0.0787)	5.5751	21.47%	60.80%	0.1621	10	-0.2933 (-0.7414, 0.1549)	7.5107	15.92%	85.33%	0.1997	12	0.0186 (-0.0574, 0.0946)	0.9357	0	11.44%	0.6311
Age	19	1.0050 (0.2696, 1.7404)	5.7148	29.31%	88.02%	0.0074	11	0.5045 (-0.1970, 1.2061)	5.5592	21.92%	59.53%	0.1587	11	0.8773 (-0.4442, 2.1989)	7.6478	8.59%	92.21%	0.1932	12	0.1060 (-0.1703, 0.3824)	1.5466	0	17.20%	0.4520

Model	TC						TG						BMI						Fibroscan CAP score					
No covariates	N	Coeff. (95% CI)	τ^2	Adj. R ^{2}%	I^2	p Value	N	Coeff. (95% CI)	τ^2	Adj. R ^{2}%	I^2	p Value	N	Coeff. (95% CI)	τ^2	Adj. R ^{2}%	I^2	p Value	N	Coeff. (95% CI)	τ^2	Adj. R ^{2}%	I^2	p Value
Univariate																								
CFU	9	0.0001 (-0.0001, 0.0004)	11.6302	0	77.08%	0.3578	12	0.0004 (-0.0000, 0.0008)	10.6041	17.89%	83.22%	0.0770	10	0.0001 (-0.0001, 0.0003)	0.7947	17.22%	83.66%	0.1983	8	0.0001 (-0.0001, 0.0003)	0.8703	0	88.13%	0.2644
ALT baseline	14	-0.1836 (-0.5296, 0.1623)	12.7425	0	83.84%	0.2982	17	-0.3876 (-0.8333, 0.0581)	9.9583	23.79%	81.67%	0.0883	13	0.0080 (-0.0146, 0.0306)	0.8119	0	84%	0.4873	8	0.0237 (-0.0206, 0.0680)	0.8632	0.20%	84.18%	0.2945
AST baseline	13	-0.2147 (-0.5626, 0.1333)	12.3511	6.03%	92.77%	0.2266	16	-0.6443 (-1.0299, -0.2588)	6.1253	73.36%	50.50%	0.0011	13	0.0117 (-0.0100, 0.0334)	0.7850	3.95%	82.14%	0.2906	8	-0.0383 (-0.1001, 0.0235)	0.8208	9.75%	82.88%	0.2243
Age	12	1.8456 (1.0162, 2.6750)	5.3212	83.09%	35.90%	<.0001	15	1.8879 (0.6274, 3.1484)	8.1907	57.11%	50.81%	0.0033	13	0.0164 (-0.0836, 0.1160)	0.8316	0	87.89%	0.7482	8	0.0049 (-0.1709, 0.1807)	0.9688	0	90.41%	0.9565

ALT: Alanine Aminotransferase; AST: Aspartate Aminotransferase; BMI: Body Mass Index; CFU: colony-forming unit; FBG: Fasting Blood Glucose; GGT: Gamma-Glutamyl Transferase; TC: Total Cholesterol; TG: Triglycerides
